# Pathologies at the gateway: exploring the link between nucleoporins and inherited diseases

**DOI:** 10.1007/s00018-026-06220-2

**Published:** 2026-04-29

**Authors:** Daniela A. Braun, Ramona Jühlen, Vanessa Krausel, Wolfram Antonin

**Affiliations:** 1 Department D of Internal Medicine, University Hospital of Münster, Münster, Germany; 2 Institute of Biochemistry and Molecular Cell Biology, Medical School, RWTH Aachen University, Aachen, Germany

**Keywords:** Steroid resistant nephrotic syndrome, Monogenic disease, Nuclear pore complex, Nuclear transport, Neurological diseases

## Abstract

**Abstract:**

Nuclear pore complexes serve as essential gatekeepers of the nuclear envelope, playing crucial roles in regulating transport across the nuclear envelope and maintaining compartmentalization between the nucleus and cytoplasm. While they are fundamental to all nucleated cells, the nucleoporins that make up these complexes are associated with various inherited diseases, often affecting specific cells, tissues, or organs. In this overview, we describe the clinical features, summarize genotype-phenotype correlations at the level of individual genes and specific alleles, and relate this information to insights from cellular biology regarding nucleoporins to illuminate potential disease mechanisms. Our aim is to include significant clinical perspectives that are frequently overlooked in standard cell biology reviews, while ensuring accessibility for readers without a medical background. At the same time, we hope to provide valuable insights for geneticists and clinicians interested in the discussed pathologies, but may have limited background in molecular cell biology.

**Summary statement:**

Mutations in nucleoporin-encoding genes, the proteins that form nuclear pore complexes, are associated with various hereditary diseases. We summarize our emerging knowledge to connect clinical manifestations with insights from cell biology.

**Supplementary Information:**

The online version contains supplementary material available at 10.1007/s00018-026-06220-2.

## Introduction

Eukaryotic genomes are protected and separated from the cytoplasm by a nuclear envelope, a double-membrane structure that forms a barrier between the nucleoplasm, where gene transcription and primary transcript processing occur, and the cytoplasm, where protein translation takes place. This barrier requires highly efficient transport gates that enable selective and effective exchange of metabolites and macromolecules, including proteins and RNA-protein complexes, between the nucleoplasm and cytoplasm. These transport gates consist of nuclear pore complexes (NPCs), large macromolecular structures that create circular openings in the nuclear envelope formed by the fusion of the two nuclear membranes. They prevent most macromolecules from freely passing by establishing a permeability barrier within the pore, while also allowing the controlled import and export of specific proteins and RNA-protein complexes through nuclear transport receptor (NTR)-mediated pathways. Additionally, NPCs and their protein constituents, nucleoporins, can interact with particular genes and influence gene expression in a transport-independent manner.

Interestingly, there are different connections between nucleoporins and human disease. In particular, genetic variants affecting various nucleoporins have been linked to numerous monogenic diseases. Furthermore, chromosomal aberrations in nucleoporin-encoding genes that produce oncogenic fusion proteins have been identified as drivers of various human cancers. Autoantibodies targeting nucleoporins may cause autoimmune liver disease, and age-related deterioration of NPC may contribute to neurodegenerative disorders.

### Nucleocytoplasmic transport

**Fig. 1:**
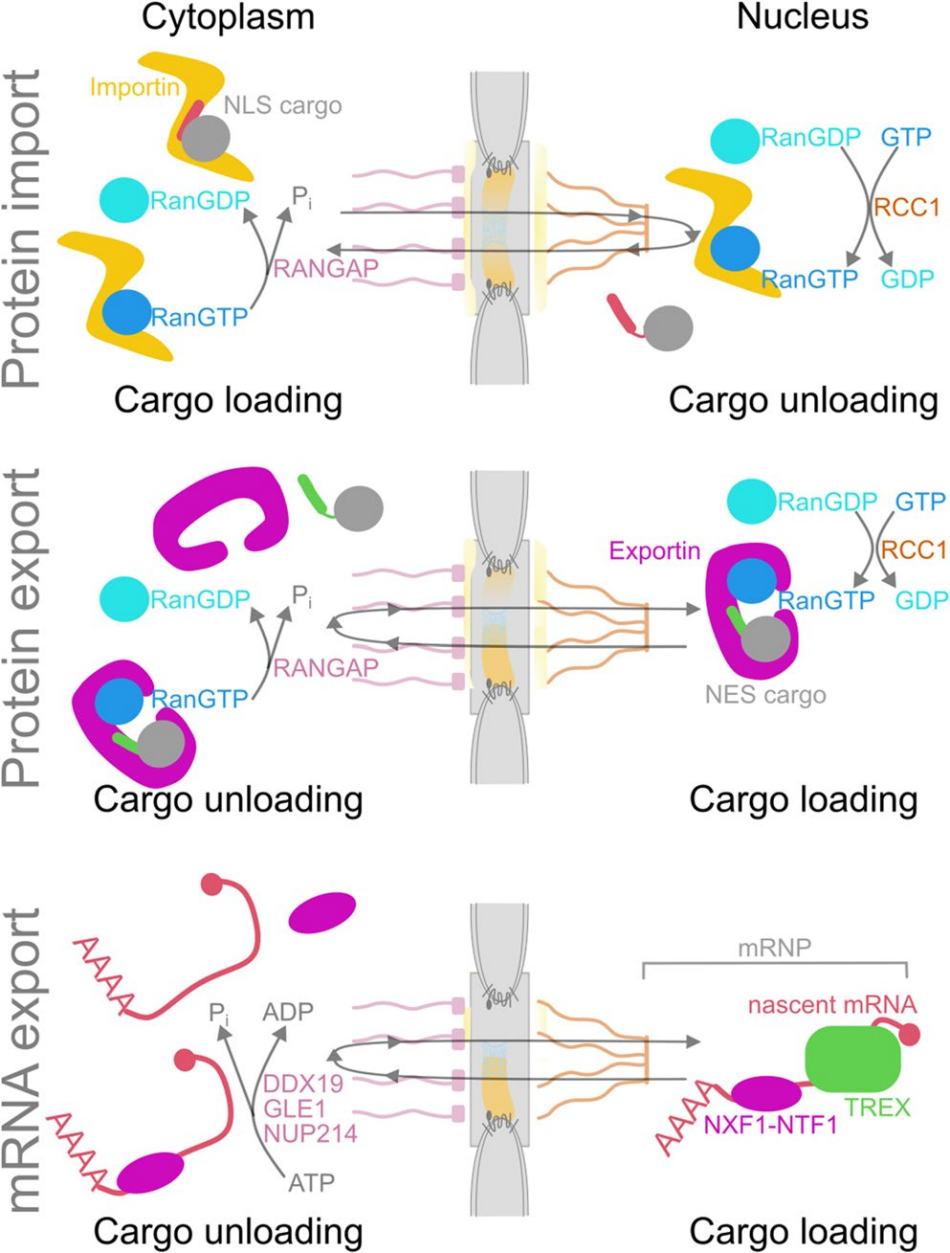
Scheme of nuclear-cytoplasmic transport: Nuclear protein import involves importins, which bind to their cargo through a nuclear localization signal (NLS) in the cytoplasm. After entering the nucleus, RanGTP binds to importins, promoting the release of the cargo. RanGTP is produced in the nucleus from RanGDP by the guanine nucleotide exchange factor RCC1. Hydrolysis of RanGTP occurs on the cytoplasmic side of the nuclear pore complex by the RanGTPase activating protein RANGAP, leading to the disassembly of the importin-Ran complex and ensuring directional transport. For nuclear export, cargo recognized by their nuclear export signals (NES) forms a complex with exportins and RanGTP in the nucleus. When this complex reaches the cytoplasm, it dissociates due to hydrolysis of RanGTP, releasing the cargo. Notably, mRNA export typically does not require RanGTP directly; instead, it relies on ATP-utilizing RNA helicases on the cytoplasmic site of the nuclear pore complex (such as DDX19 and GLE1, recruited by NUP214) and RNA export factors (like TREX and NXF1-NTF1) that bind within the nucleus

NPCs facilitate the passage of molecules primarily through two mechanisms: passive diffusion, which is effective for small molecules and becomes less efficient as molecular mass increases, and receptor-mediated translocation involving selected cargos and NTRs or karyopherins [1]. NTRs travel between the cytoplasm and nucleus, binding cargo on one side and releasing it on the other, thus facilitating translocation across the NPC permeability barrier. Transport classifies NTRs as importins or exportins, although some can function in both directions. Importins recognize nuclear localization signals on cargos and carry them into the nucleus, while exportins bind cargos with nuclear export signals in the nucleus and move them into the cytoplasm (see Fig. 1).

Importins and exportins pass through NPCs in both directions, and similarly, importin-cargo and exportin-cargo complexes could follow the same pattern. The small GTPase Ran controls the directionality of this transport. Like many small GTPases, Ran relies on auxiliary factors to cycle between GDP and GTP states. It’s guanine nucleotide exchange factor RCC1, which binds to chromatin and promotes the exchange of GDP for GTP in the nucleus, thereby creating a high concentration of RanGTP. Conversely, Ran’s GTP hydrolysis mainly occurs in the cytoplasm or on the cytoplasmic side of NPCs, induced by RANGAPs, the GTPase-activating proteins. Accordingly, Ran in the cytosol is mainly loaded with GDP.

After the importin-cargo complex forms in the cytoplasm and enters the nucleus, Ran in its GTP-bound form displaces the cargo from importin, leading to cargo release. The importin-RanGTP complex then moves through NPCs into the cytoplasm, where GTP hydrolysis causes it to dissociate. The freed importin can then participate in another import cycle. For export, a trimeric complex of the cargo, exportin, and RanGTP forms in the nucleus. After translocating through the NPC, this complex dissociates in the cytoplasm upon the hydrolysis of GTP by Ran. Aside from mRNA export, the direction of nucleocytoplasmic transport depends on the RanGTP/RanGDP gradient across the nuclear envelope as an energy source. mRNA export, in contrast, is ATP-dependent and mediated by RNA helicases like DDX19, which regulate NPC translocation and dissociation of capped and spliced mRNA-containing RNPs from NTRs, allowing their translation [2].

### NPC structure

**Fig. 2:**
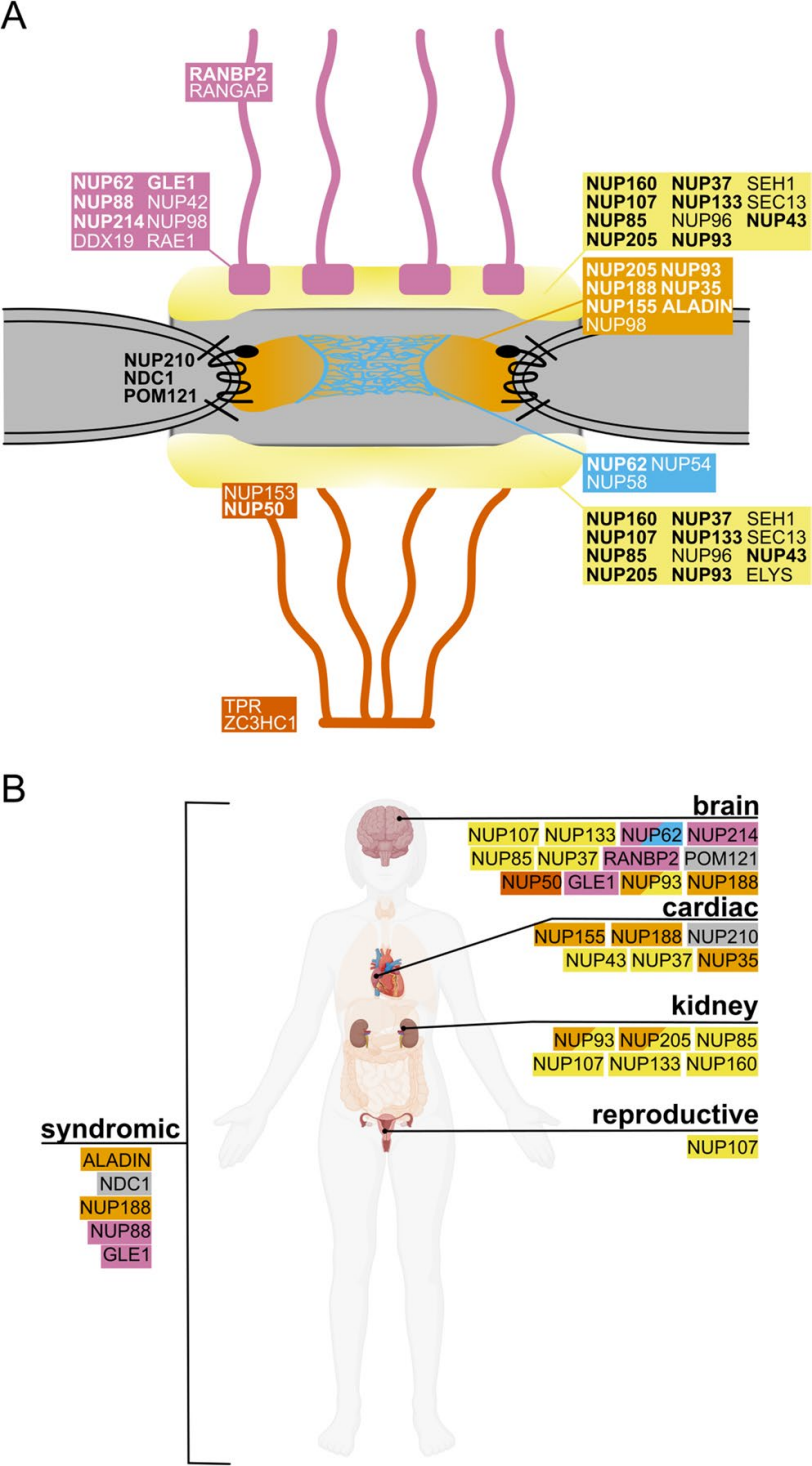
Structural Arrangement of Nucleoporins in Nuclear Pore Complexes and Their Role in Inherited Monogenic Diseases. **A** Structural overview of the nuclear pore complex (NPC). Transmembrane nucleoporins integrated into the pore membrane are shown in black. The cytoplasmic and nucleoplasmic rings, represented in yellow, form a structural framework that connects various components on both sides of the NPC. The inner ring, illustrated in orange, provides stability and support as part of the central core of the NPC. Phenylalanine-glycine-repeat nucleoporins form the central transport channel, are depicted in blue. Extending from the nucleoplasmic side is the nuclear basket, shown in vermilion; this structure plays a crucial role in regulating transport into and out of the nucleus. Cytoplasmic filament structures are represented in pink, facilitating interactions with cytoplasmic transport machinery. Nucleoporins associated with monogenic diseases discussed in this review are marked in bold. **B** Overview of various inherited monogenic diseases caused by mutations in genes encoding nucleoporins, highlighting the affected organs. Each condition is linked to specific nucleoporins

The NPC is one of the largest supramolecular assemblies in eukaryotic cells, with a mass of approximately 120 MDa and comprising roughly 1,000 individual proteins in vertebrates. It is formed by approximately 35 different nucleoporins (Fig. 2A), which, due to the eightfold symmetry along the nucleocytoplasmic axis of NPCs, are present in multiples of eight [3]. The symmetric core across the nuclear envelope plane includes an inner ring located within the pore channel, flanked by two outer rings that rest on the membrane surfaces. Nucleoporins can be generally divided into two classes. Scaffold nucleoporins contain folded domains and form a cylindrical NPC backbone structure around a central channel. Intrinsically disordered nucleoporins, mainly containing multiple phenylalanine-glycine (FG) repeats and accordingly referred to as FG-nucleoporins, primarily line the scaffold and extend into the central channel, where they form the diffusion barrier but can also interact with cargo complexes and allow their passage. Three integral pore membrane domain proteins, POM121, NDC1, and NUP210, have both pore-facing and nuclear envelope lumen-facing domains. Large asymmetric cytoplasmic filaments and nuclear basket structures are attached to this symmetric core, and serve as molecular docking stations for import and export complexes.

The inner ring contains multiple copies of scaffold nucleoporins NUP205, NUP188, NUP155, NUP93, and ALADIN, connected by unstructured linkers NUP98 and NUP35, also known as NUP53 [3, 4]. It attaches to the membrane, including the membrane nucleoporins, and, along with NUP93, forms the binding site for the NUP62 complex, which includes NUP62, NUP58, and NUP54. The NUP62 complex plays a significant role in forming the diffusion barrier in the center of the channel, but other nucleoporins like NUP98 also contribute to this barrier. The nuclear and cytoplasmic outer rings are primarily formed by 16 copies of the NUP107-NUP160 complex, also known as the Y-complex due to its overall shape [5]. In vertebrates, the NUP107-NUP160 complex consists of nine nucleoporins, NUP160, NUP133, NUP107, NUP96, NUP85, NUP43, NUP37, SEC13 and SEH1. Additional copies of NUP93 and NUP205, which also make up a considerable part of the inner ring, comprise these outer rings, and on the nuclear side, additionally, ELYS/MEL28 is also involved [4, 6].

The eight cytoplasmic filaments extend roughly 60 nm into the cytoplasm. Each consists of a RANBP2 homopentamer, which provides numerous Ran-binding sites [7]. It also recruits the Ran-GTPase activating protein 1 (RANGAP1), which aids in disassembling export complexes. At the base, a second module comprises a subcomplex that includes NUP214, NUP88, NUP42, RAE1, GLE1, additional copies of NUP62 and NUP98, and the DEAD-box helicase DDX19, all of which play crucial roles in mRNA export.

At the nuclear face of the NPCs, up to 120-nm basket-like structures observed by electron microscopy are formed by NUP153, NUP50, TPR, and ZC3HC1. These basket-like protrusions likely originate from the extensive coiled-coil domains of TPR and its connector ZC3HC1 [8], while NUP153 and NUP50 are probably located at the base of the nuclear basket, interacting with the nuclear ring structure. The nuclear basket is involved in excluding chromatin from the NPC’s periphery, selecting export-ready mRNAs and preribosomal particles, protein quality control, and gene tethering. Since NUP153 and NUP50 provide Ran binding sites, they also serve an auxiliary role in protein import into the nucleus.

### NPC assembly

Cells preserve NPC numbers, composition, and function at steady state through two distinct assembly pathways: rapid NPC reassembly after mitosis and a much slower de novo NPC biogenesis during interphase into the intact nuclear envelope.

During mitotic NPC assembly, late anaphase and telophase involve the mitotic endoplasmic reticulum interacting with decondensing chromatin to restore the nuclear envelope. At the same time, NPCs are reassembled gradually, starting at the chromatin surface, using existing building blocks and integrating into small openings in the reforming nuclear membranes [9, 10]. After the formation of the central ring scaffold, the assembly of the NUP62-complex in the central channel enlarges the pore to its final size and establishes the diffusion barrier [11]. Then, the assembly of cytoplasmic filaments and nuclear basket structures finishes the reassembly, allowing the NPC to recover its full transport function [9].

Interphase NPC assembly occurs within the intact two-membrane nuclear envelope, requiring fusion of both the inner and outer nuclear membranes [12]. This assembly follows an ‘inside-out’ mechanism, where new NPCs protrude through the nuclear envelope by evaginating the inner nuclear membrane, which then fuses with the outer membrane to form the pore through a process that is not fully understood [13]. The entire process takes hours, beginning with the incorporation of the transmembrane nucleoporin POM121 and the nuclear basket nucleoporin NUP153, followed by the addition of outer and inner ring components, and culminating in the assembly of cytoplasmic filaments. In non-dividing cells, this is the only method used to maintain or increase the number of NPCs. Although NPCs are often seen as static and durable, the stability of individual nucleoporins within an NPC varies greatly. FG-nucleoporins may stay within NPCs for seconds, while scaffold nucleoporins like NUP205 or NUP93, especially those of the inner ring, are very long-lasting and might not be exchanged during the cell’s lifetime [14, 15].

Although NPCs play an essential role in maintaining the compartmentalization between the nucleus and cytoplasm in all nucleated cells, genetic variants in nucleoporin-encoding genes can lead to phenotypes that are often specific to certain tissues or cell types, resulting in various diseases. In other cases, genetic variants in nucleoporin genes manifest in multiple cell types, leading to multi-organ disease phenotypes. In this review, we will examine our current understanding of how genetic variants in nucleoporins influence NPC function and explore the implications of these mutations across different diseases (Fig. 2B).

## Nucleoporins in cancer and autoimmune diseases

Beyond monogenic variants involved in hereditary human disorders, which will be the focus of our review, there are also other mechanisms linking nucleoporins to human diseases, most notably to cancer, either through overexpression in malignant tissues or due to the formation of chromosomal translocations that lead to oncogenic fusion proteins (reviewed [16]), and to autoimmune diseases through the formation of nucleoporin-targeting antibodies.

Examples of nucleoporins linked to cancer include NUP88, which is overexpressed in several cancer types, most notably various carcinomas, but also in tumors of other origins such as mesotheliomas, gliomas, sarcomas, and lymphoreticular tumors [17, 18]. In breast cancer, the degree of NUP88 overexpression correlates with invasive cancer types, tumor grade, high proliferation rates, and high expression of oncogenes, indicating that it may serve as a marker for tumor aggressiveness [19]. NUP88 is part of the base of the cytoplasmic filament and forms a complex with the FG-repeat nucleoporin NUP214. The NUP88-NUP214 complex directly binds to the major NTR Exportin-1/CRM-1 [20] and is involved in nuclear export [21]. Although the exact mechanism linking the NUP88 overexpression to cancer cell transformation remains unknown, it may involve the nucleocytoplasmic distribution and nuclear activity of prooncogenic transcription factors like NF‑κB [22]. Another example is elevated NUP210 expression, which is associated with increased metastatic potential and poorer prognosis in breast cancer [23] and acute myeloid leukemia (AML) [24].

NUP214, also known as CAN, is a prominent example of a chromosomal rearrangement in leukemia that results in a fusion protein of NUP214 with the chromatin-binding proteins DEK or SET. NUP214 is an FG-repeat nucleoporin located on the cytoplasmic side of the NPC that plays a key role in mRNA export. The oncogenic fusion protein retains the C-terminal two-thirds of NUP214 fused to the full Dek or Set protein, which keep their chromatin-modifying properties [25], leading to altered transcriptional profiles in the presence of NUP214 fusion proteins [26]. On the other hand, due to the retained C-terminal part of NUP214, which can still bind Exportin-1/CRM-1, the fusion protein may also interfere with nuclear transport [27]. A third chromosomal translocation fuses NUP214 to the ABL1 gene locus, generating an active Abl tyrosine kinase that is no longer controlled by its original promoter and can be detected in approximately 6% of patients with T-cell acute lymphoblastic leukemia [28]. Tyrosine kinase inhibitors specifically directed against the overactive Abl kinase can be used for targeted therapy in these patients. Chromosomal rearrangements involving NUP98 are even recognized as a genetically defined subtype of AML associated with poor prognosis and chemotherapy resistance [29]. NUP98 is also an FG-repeat nucleoporin essential for the NPC permeability barrier [30]. A fusion of the N-terminal half of NUP98 with the C-terminal part of the HOXA9 transcription factor, leading to altered transcriptional programs, was the first identified NUP98 fusion protein in AML [31, 32]. Subsequently, more than 40 different fusion partners, each associated with distinct AML differentiation types, have been identified [29]. Most NUP98 fusion partners are transcription factors [16], suggesting that similar molecular mechanisms underlie the diverse NUP98 fusion proteins. TPR is a giant nucleoporin at the nucleoplasmic site of NPCs that serves as a major docking station for mRNA export. TPR is also involved in oncogenic translocations, including one to the proto-oncogene MET in gastric cancer [33]. MET is a receptor tyrosine kinase for hepatocyte growth factor, and the oncogenic fusion leads to uncontrolled, constitutively active downstream signaling of, e.g., MAP kinases, which drive oncogenic transformation, invasive behavior, and metastasis [34]. Because of the strong connection between certain nucleoporins and cancer development, tumor progression, and prognosis, the NPC and its properties are also discussed as a potential targets for cancer therapy [35].

Besides their link to cancer, nucleoporins are also implicated in autoimmune diseases [36], a group of disorders in which, for reasons that are incompletely understood, loss of immunotolerance leads to the formation of autoreactive antibodies targeting specific cell types, individual organs, or multiple organ systems. Phenotypes resemble those of infectious diseases, with fever, systemic inflammation, and progressive organ damage caused by inflammatory cell infiltration. If untreated, chronic tissue inflammation ultimately leads to scarring and irreversible loss of organ function. Autoimmune disorders frequently manifest at an early age, are more prevalent in females, and commonly require lifelong immunosuppressive therapy. Although the causality of nucleoporin-targeting autoantibodies and their detailed pathogenic mechanisms remains unknown, it has been suggested that autoantibody formation results from molecular mimicry between human NPC proteins and components of various viruses [37]. NPC autoantibodies have been sporadically detected in different autoimmune diseases, including connective tissue disorders like myositis [38], systemic lupus erythematosus [39], and mixed connective tissue disease [40]. Their clinical significance is most clearly established in primary biliary cholangitis, where progressive autoinflammatory destruction of cholangiocytes, the epithelial cells lining the bile ducts, ultimately leads to liver cirrhosis and the need for transplantation. In primary biliary cholangitis, 22–32% of patients have antibodies against NUP210, while 22–31% have antibodies targeting NUP62 [36]. NUP210, also known as GP210, is a transmembrane nucleoporin, and NUP62 is an FG-nucleoporin that, along with its interaction partners NUP58 and NUP54, forms the main part of the gel-like matrix within the central NPC channel. Additionally, NUP62 also participates in a different NPC subcomplex, interacting with NUP214, NUP98, NUP88, NUP42, RAE1, and GLE1 at the base of the cytoplasmic filaments. Some studies suggest an association between NPC autoantibodies and poor clinical outcomes [41], but the full clinical significance of these antibodies remains to be determined.

## Nucleoporin-linked hereditary disorders

### Nucleoporins in heart diseases

Several nucleoporins have been associated with various cardiovascular diseases, including arrhythmias, dilated cardiomyopathies, ischemic cardiomyopathy, congestive heart failure, valve and vascular disorders, as well as heterotaxy and *situs inversus* [42]. Some of these diseases are linked to altered expression of nucleoporins, but we will focus on the hereditary forms.

#### NUP155 and NUP37 in atrial fibrillation

Atrial fibrillation (AF; MIM #608583) is the most prevalent age-related cardiac arrhythmia, with its occurrence expected to rise significantly amid global demographic shifts [43, 44]. It involves rapid, irregular atrial contractions, which may impair cardiac function and cause symptoms such as palpitations, shortness of breath, dizziness, and fatigue. AF is a major risk factor for ischemic stroke, contributing substantially to morbidity and mortality. Treatment of AF involves continuous anticoagulation to prevent the formation of blood clots within the dysfunctional, fibrillating atrium and consecutive thromboembolic events like strokes. Furthermore, antiarrhythmic pharmacotherapy is used to restore a proper sinus rhythm and prevent tachycardia. Additionally, invasive procedures are increasingly performed to ablate dysfunctional parts of the atrium and restore the heart’s normal electrical properties. While most cases are linked to modifiable risk factors like hypertension, diabetes, and obesity, or to structural heart defects such as atrial dilation, valvular disease, and congestive heart failure, emerging evidence indicates a genetic component in a subset of patients. Beyond polygenic risk factors, also monogenic variants in genes encoding ion channels have been implicated in AF pathogenesis [45]. In addition, cytoskeletal gene variants have been implicated as causative for AF [46].

Genetic variants in a highly conserved residue of *NUP155* have been linked to both familial and neonatal AF, indicating that dysfunction of NPCs may contribute to AF development. The p.Arg391His variant has been associated with early-onset AF and sudden cardiac death [47]. While carriers heterozygous for this genetic variant have been reported to exhibit normal phenotypes without cardiac disease, offspring carrying the variant in the homozygous state died perinatally due to arrhythmogenic impairment. Another heterozygous variant in *NUP155* gene, p.L503F, was found in a 26-year-old male who died from a sudden unexplained nocturnal death presumably linked to AF [48]. The disease-causing potential of other *NUP155* variants remains uncertain [49, 50].

NUP155 is a highly conserved nucleoporin essential for NPC function, playing key roles in nucleocytoplasmic transport, nuclear envelope formation, and NPC assembly [51, 52]. It is located in the NPC’s inner ring [53] and has a structural domain organization consisting of a N-terminal β-propeller and a C-terminal α-solenoid. In addition to its structural function, NUP155 is vital for embryonic development and facilitates mRNA export, including HSP70 mRNA, through interactions with GLE1 and NUP42, also known as CG1 or NUP2L [54, 55].

While homozygous *Nup155* knockout mice are embryonically lethal, heterozygous mice show the AF phenotype and early cardiac death. Functional studies indicate that both, the p.Arg391His mutation and decreased NUP155 levels impair the nuclear export of Hsp70 mRNA and the import of Hsp70 protein. The p.Arg391His variant was initially thought to disrupt NUP155’s localization at the NPC and to impair nuclear envelope permeability [47]. This dysfunction was proposed to interfere with nucleocytoplasmic transport, subsequently impacting gene expression and consecutively functional properties of cardiac cells. Nonetheless, experiments using in vitro assembled NPCs in *Xenopus* egg extracts and transfections of GFP-tagged wild-type and mutant NUP155 into mouse fibroblasts failed to confirm the mislocalization of the p.Arg391His mutant away from NPCs [56, 57]. NUP155 is a highly stable nucleoporin within the NPC [14], and the p.Arg391His variant does not affect its mobility [57], indicating that other functions of NUP155 might be responsible for the cardiac phenotypes observed.

Besides its NPC functions, NUP155 also plays non-canonical roles. It has been linked to the p53 pathway by regulating the cyclin-dependent kinase inhibitor p21, indicating a function in cellular stress responses and DNA repair [58]. Additionally, NUP155 can bind chromatin directly, resulting in changes in chromatin positioning and gene expression in a neonatal rat ventricular model of cardiac hypertrophy, partly through interactions with histone deacetylase HDAC [59]. Similar mechanisms are observed in budding yeast, where the NUP155 homolog, Nup170, interacts with the silencing protein Sir4 [60]. NUP155 C-terminus interacts with HDAC4 [59], yet the AF-associated variants p.Arg391His and p.Leu503Phe are found within the N-terminal β-propeller domain. Since NUP155’s inherent autoinhibition involves interactions between its N- and C-terminal regions [56], HDAC4 binding to the C-terminal domain could affect NUP155’s self-inhibition. However, direct evidence for this has yet to be established.

Disruption of one *Nup155* allele in a pro-arrhythmogenic mouse embryonic stem cell model of cardiogenesis leads to widespread transcriptomic changes, including non-coding RNA changes, which affect cardiogenic signaling modules and stem cell pluripotency [61, 62]. Interestingly, when these cells are differentiated into beating embryoid bodies, they form contractile foci containing cardiomyocytes that replicate the electrophysiological deficits seen in AF. Therefore, changes in gene expression likely cause the NUP155-associated AF phenotype, probably through epigenetic mechanisms, although a role for nuclear transport cannot be excluded [47].

Whole-exome sequencing in patients with severe cardiovascular disease revealed a heterozygous nonsense variant of *NUP37*, p.Ser109*, in a patient with clinical AF and sudden cardiac death [63]. NUP37 is a component of the NUP107-NUP160 subcomplex, also known as the Y-complex, which is symmetrically positioned on both sides of the nuclear membrane. In metazoans, the Y-complex is composed of two concentric rings, each with eight members, forming a significant part of the cytoplasmic and nucleoplasmic rings [5]. NUP37 is localized to the short arm of the Y-complex, where it forms a tight complex with NUP160 and stabilizes the interaction between domains of NUP160, further supporting the overall architecture of the short arm. This places NUP37 close to the membrane-proximal surface of the Y-complex, orienting it toward the nuclear envelope and likely facilitating the integration of the Y-complex with transmembrane nucleoporins. Morpholino-mediated reduction of Nup37 expression in a zebrafish model resulted in the development of arrhythmias, heart failure, and abnormalities in the heart chambers, like an enlarged or malformed atrium [63].

Interestingly, several AF-associated lamin A/C (LMNA) mutations have been identified [64, 65]. One of these disrupts the interaction between lamin A/C and NUP155 and is thought to impair nucleocytoplasmic transport [66]. It will be interesting to see whether the *NUP155* variants linked to AF also impact this interaction. If so, it could imply a connection between NUP155 and the nuclear skeleton as factors in the development of AF. Additionally, it is noteworthy that Nesprin 2 mutations have also been associated with AF [46, 67].

#### Nucleoporins linked to developmental defects in left-right patterning and congenital heart disease

Several nucleoporins have been implicated in the developmental processes that ensure the correct positioning of the organs within the body. Defects in left-right patterning, such as *situs inversus* and heterotaxy, are rare, occurring in approximately 1 in 1,000 births. *Situs inversus totalis* is a congenital condition where the organs are arranged in a complete mirror-image reversal of their normal positions, which, however, typically does not lead to severe complications. In contrast, patients with heterotaxy have major visceral organs distributed abnormally without mirror symmetry, often accompanied by developmental malformations, primarily congenital heart defects (CHDs). The spectrum of congenital heart defects ranges from conditions that are incompatible with survival and require immediate surgical correction after birth, to defects that impair blood oxygenation and limit physical activity yet allow normal development, and finally to defects that do not require immediate treatment but increase the risk of valvular heart disease or congestive heart failure in adulthood.

Genetic findings link distinct structural nucleoporins to the establishment of left-right asymmetry. NUP93 forms stable complexes with NUP205 and its paralogue NUP188, which are present in 48 copies in the inner NPC ring but are also found in the nucleoplasmic and cytoplasmic ring structures, thus constituting a major structural part of the NPC. This link between these nucleoporins and left-right asymmetry was initially indicated by the discovery of a *NUP188* gene duplication in a patient with heterotaxy and CHD [68]. It might be speculated that overexpression of NUP188 changes the balance of the two orthologues NUP205 and NUP188, which both tightly interact with NUP93 [69], in cells. Surprisingly, bi-allelic truncating *NUP188* variants also caused a syndromic developmental phenotype in six affected individuals, including congenital cataracts, hypotonia, prenatal ventriculomegaly, white matter abnormalities, hypoplastic corpus callosum, CHD, and central hypoventilation [70], suggesting that both, increased expression as well as loss of NUP188 impair embryonic development. Genetic variants in the *NUP188* paralogue *NUP205* (p.Thr1044Met and p.Pro1610Arg), and the transmembrane nucleoporin *NUP210* have been observed in individuals with CHD [71]. Corresponding to the human phenotype, overexpression as well as knockdown of *nup188* in Xenopus [72, 73] and *nup205* knockdown in zebrafish [71] or Xenopus [73] disrupted cardiac left-right patterning. In addition, the downregulation of *nup93* in Xenopus showed similar left-right patterning defects; however, in humans, genetic variants in *NUP93* have not yet been linked to CHD. GLE1, a nucleoporin at the base of the cytoplasmic filaments that will be discussed in more detail later, has been associated with left-right patterning in zebrafish [74]. Also, here, mutations in the human proteins linked to CHD have not been reported so far.

Defects in left-right patterning are often linked to defective cilia function as cilia in developing embryos generate a leftward body fluid flow that signals the body’s left and right sides. Indeed, a sub-fraction of NUP188, NUP205, and NUP93 localizes at the cilia base and centrosomes, and their knockdown causes cilia loss in Xenopus embryos [72, 73, 75]. Interestingly, in this system, loss of NUP188 can be compensated by NUP205 overexpression and vice versa [73]. Notably, of the two CHD-variants in *NUP205*, the p.Pro1610Arg variant cannot replace the wildtype protein, while p.Thr1044Met can. Downregulation of NUP205 in human retinal pigment epithelium impacts cilia length and density [71].

It has been proposed that the nucleoporins that localize to the ciliary base form a ciliary pore complex similar to the NPC, functioning as a selective gate that controls access to the cilium [76]. However, super-resolution imaging of NUP188 at the ciliary base is largely incompatible with the model of an NPC-like ring [72, 77]. Rather, NUP188 and likely other nucleoporins at the ciliary base have a unique function on this side, and at least NUP188 has been shown to play a role in centrosome duplication [77]. Still, the precise action remains to be elucidated. Similarly, the roles of GLE1 and NUP210 in left-right patterning, as well as their potential connection to cilia function, remain unclear.

### NUP107 in XX-ovarian dysgenesis

Another defect in a developmental program involving a structural nucleoporin is XX-ovarian dysgenesis (XX-OD). XX-OD (MIM #618078) is a rare, genetically diverse disorder characterized by underdeveloped, dysfunctional ovaries. This leads to a lack of spontaneous puberty, primary amenorrhea, meaning the absence of menstruation, uterine hypoplasia, and hypergonadotropic hypogonadism [78]. Circulating levels of follicle-stimulating hormone and luteinizing hormone are both decreased, indicating ovarian dysfunction. Confirmatory genetic testing, especially chromosomal analysis via karyotyping, is essential to verify the presence of XX chromosomes and to rule out conditions like Turner syndrome (XO) or 46,XY disorders of sex development [79].

A subset of XX-OD cases has been associated with NUP107. The first identified causative variant in the *NUP107* gene (p.Asp447Asn) was described in five female cousins from a Palestinian consanguineous family [80]. All male relatives showed normal pubertal development, and married men had multiple children, suggesting that the variant’s effects are female specific. Another variant in *NUP107* (p.Arg355Cys) was independently linked to XX-OD in a non-consanguineous Mexican family with two daughters diagnosed with primary ovarian insufficiency [81].

NUP107 is like NUP37 a component of the NUP107-NUP160 subcomplex, which dominates the cytoplasmic and nucleoplasmic ring structures [5]. NUP107, together with NUP133, forms the stalk of the Y-complex and is essential for its stability. The NUP107-NUP160 complex plays a crucial role in mitotic and interphase NPC assembly [82–85] and mRNA export [86]. Furthermore, members of the NUP107 complex are actively involved in mitosis, helping regulate kinetochore assembly [87, 88]. The Y-complex’s involvement in numerous vital cellular processes highlights its importance in maintaining cellular homeostasis.

Both, the p.Asp447 and p.Arg355 residue are involved in conserved salt bridge interactions [80, 81], which may weaken the tight, tail-to-tail interface between the C-terminal domains of NUP107 and NUP133, essential for the stability of the Y-complex. When modeling the p.Asp447Asn mutation in *Drosophila melanogaster*, the protein localized correctly at the nuclear envelope [80]. Female flies exhibited defective oogenesis and significantly reduced fertility [80, 89], while male flies were unaffected. Similarly, a homozygous *nup107* p.Arg355Cys knock-in in female mice led to decreased fertility [81]. Different explanations have been proposed for why mutations specifically affect female oocyte maturation. One hypothesis links this to the meiotic DNA damage response; however, direct evidence for this connection is currently lacking [80]. Alternatively, changes in gene expression might be responsible [89]: transcriptome analysis identified the sex-determination gene doublesex (dsx) as a primary target downstream of NUP107 in *Drosophila*. The loss of Dsx mimics the phenotypes seen with NUP107 loss, which is also similar to the effects of dysregulated bone morphogenetic protein (BMP) signaling. Conversely, overexpression of Dsx was able to rescue the phenotypes caused by NUP107 deficiency [89]. Lastly, since the Y-complex plays a role in kinetochore function, dysfunction disrupting spindle-chromatin interaction cannot be ruled out, which might impact meiotic and mitotic cell divisions.

### GLE1 in LCCS1 and CAAHD

Lethal congenital contracture syndrome 1 (LCCS1, MIM #253310) is a severe condition present before birth, belonging to a diverse group of diseases called arthrogryposis [90]. Arthrogryposis affects approximately 1 in 3,000 births worldwide and is primarily characterized by multiple joint deformities, or contractures, at birth [91]. Fetuses with LCCS1 typically show joint contractures, early loss of voluntary muscle movements, micrognathia (a small jaw), incomplete lung development, and muscle wasting [90]. These fetuses also exhibit a distinct neuropathological pattern, characterized by a significant loss of the ventral spinal cord, including the anterior horn motor neurons and the ventral and lateral columns. In contrast, the sensory nuclei and dorsal columns are less affected. LCCS1 leads to neonatal death.

Most LCCS1 patients have a homozygous splice site variant in the *GLE1* gene, resulting in a three-amino-acid insertion into the coiled-coil domain of the GLE1 protein [92]. While heterozygotes typically show no phenotype, compound heterozygotes carrying this variant along with additional amino acid substitutions in the C-terminal domain of *GLE1* exhibit a milder condition called LAAHD, which stands for lethal arthrogryposis with anterior horn cell disease. In this condition, the fetus usually survives only a short time after birth [93]. Patients with different compound heterozygous *GLE1* variants tend to survive longer, leading to the disease being renamed CAAHD, for Congenital Arthrogryposis with Anterior Horn Cell Disease (MIM # 611890).

GLE1 is a crucial factor for mRNA export and plays a role in efficient translation initiation and termination [94]. It localizes to the cytoplasmic face of the NPC via interactions with NUP42 (CG1/NUP2L) and NUP155 [54, 55]. Homozygous *gle*1−/− mutants or morpholino-mediated knockdowns in zebrafish exhibit various features of LCCS, including severe developmental defects such as motor neuron arborization abnormalities and embryonic lethality [95]. In this model, the human wild-type GLE1 can rescue the phenotype, while the splice site mutation cannot.

Mechanistically, the three-amino-acid insertion in the coiled-coil domain of GLE1 disrupts its dimerization, NPC localization, and impairs mRNA export in tissue culture cells [96]. The effects of other *GLE1* variants are less clear. GFP-fusions of variants associated with CAAHD, such as p.Arg569His, p.Val617Met, and p.Ile684Thr, do not localize to NPCs [97]. These findings suggest that they may also impact mRNA export; however, it remains unclear why these mutations, whether homozygous or combined with the heterozygous coiled-coil domain insertion, result in milder phenotypes leading to CAAHD.

### NUP88 in fetal akinesia deformation sequence

Fetal akinesia deformation sequence (FADS [MIM # 208150]) is a lethal disorder caused by defects in the neuromuscular axis. Affected fetuses are unable to move in the uterus, giving rise to the disorder’s name. Intrauterine fetal movement is a prerequisite for normal fetal development. Fetal movement restrictions cause secondary defects, including intrauterine growth retardation, major joint contractures (arthrogryposis), and other developmental anomalies like lung hypoplasia or cardiac defects. Clinical features are diverse, and affected fetuses are either lost as spontaneous abortions or stillborn. Those born alive are premature and die shortly after birth [98, 99].

FADS is genetically heterogenous and inherited in an autosomal recessive manner due to variants in genes that encode components of motor neurons, the peripheral nervous system, the neuromuscular junction, and skeletal muscle. Using exome sequencing, biallelic variants in *NUP88* have been identified as causes of FADS in two separate families: the first family carried a homozygous missense variant p.Asp434Tyr, while the second family had two compound heterozygous variants, a nonsense variant (p.Arg509*) and a single amino acid deletion variant (p.Glu634del) [100]. Zebrafish experiments demonstrated that mutant *nup88* alleles are loss-of-function variants that significantly impair locomotion behavior in zebrafish larvae, closely resembling the human phenotype [100].

NUP88 localizes to the base of the cytoplasmic filaments where it interacts with NUP214, NUP62, and NUP98 [20, 101, 102]. Additionally, a localization to the nuclear side of NPCs was suggested, where it associates with lamin A/C [103]. The NUP88-NUP214 complex regulates the nuclear export of specific proteins and pre-ribosomes alongside the nuclear export receptor CRM1 (exportin 1, XPO1) [21, 104]. Since FADS variants in *NUP88* do not significantly affect its interactions with nucleoporin complex partners, lamin A, or CRM1-dependent nuclear export in human cells, an NPC-independent role in disease pathogenesis has been proposed [100]. However, recent findings describe a homozygous splice site variant in *NUP214* (c.46–2 A > G) in a fetus exhibiting a FADS-like phenotype, including arthrogryposis and hydrops [105]. The identification of two proteins of the NUP88-NUP214 complex in the same disease suggests a shared molecular pathway, which might nevertheless be NPC-independent.

In primary human fibroblasts, knockdown of NUP88 or other FADS-related proteins impairs ciliogenesis and the general microtubule cytoskeletal network [106]. This aligns with the diverse clinical features observed in FADS patients, particularly craniofacial and skeletal defects. Additionally, the interaction between NUP88 and the focal adhesion protein paxillin, which is responsible for actomyosin contraction in skeletal muscle, was lost due to FADS-related variants in NUP88. Knockdown of NUP88 also disrupted the actin cytoskeletal network [107]. It can be hypothesized that dysfunctional NUP88 in FADS causes cytoskeletal changes, which subsequently lead to the abnormal muscle contractions observed in FADS patients.

### ALADIN in triple A syndrome

Triple A syndrome, or Allgrove syndrome, is an autosomal recessive neuroendocrine disorder marked by three main symptoms, the “classical triad”: achalasia of the cardia (where the lower esophageal sphincter has increased tone and the esophagus dilates), alacrima (absence of tear production), and adrenal insufficiency, leading to hypocortisolism, resistant to the adrenocorticotropic hormone ACTH [108]. This disorder is caused by mutations in the *AAAS* gene, which encodes the ALADIN protein, also known as Adracalin [109, 110], a component of the NPC [111–113].

To date, at least 49 pathogenic variants in the *AAAS* gene have been identified, including missense, nonsense, frameshift, and splice site mutations. These genetic changes typically cause the mislocalization of the ALADIN protein, preventing its proper incorporation into the NPC. Similarly, the complete absence of the gene results in Triple A syndrome [114]. Despite the variety of mutations, no clear genotype–phenotype correlation has been observed. Patients with the same mutation, even within the same family, can display significantly different clinical features.

Beyond the three characteristic symptoms, Triple A syndrome can present as a multisystem disorder with a highly variable clinical picture [115, 116]. Affected individuals may also show neurological features such as peripheral neuropathy, distal muscle wasting, dysarthria, which is a motor speech disorder caused by nervous system damage leading to paralysis or weakness of speech muscles, nasal speech, movement disorders, and intellectual disability. They may also experience autonomic dysfunction, including abnormal sweating, orthostatic hypotension, and pupillary abnormalities. Other phenotypes can include short stature, scoliosis, palmoplantar hyperkeratotic thickening of the palms and soles, dental caries, poor wound healing, and rarely, cleft palate or multiple nasal polyps. There is high phenotypic variability, with disease onset and severity ranging from mild, late-onset neurological symptoms to early-onset, life-threatening adrenal crises. Pediatric patients more often present with the classical triad, whereas late-onset cases may primarily show neurological involvement [117]. It has been suggested that neurological symptoms may appear in certain subgroups of patients with a milder and more chronic course of the disease [118]. Due to its rarity with an estimated prevalence of 1 in 1 million individuals, and clinical variability, the diagnosis of Triple A syndrome is often delayed or overlooked [119]. Besides the neurological and autonomic features of the disease that can only be treated symptomatically, management of Triple A patients, most importantly involves hydrocortisone replacement therapy, which needs to be dose-adjusted in stress situations such as severe illness, accidents, or intense physical activity. Patients and their guardians need to be educated about the signs of developing adrenal crisis, such as hypotonia, hypoglycemia, electrolyte imbalance, dehydration, gastrointestinal symptoms or pain, altered mental status, and ultimately, hypotensive shock. Hydrocortisone replacement must be lifelong, and patients should carry an emergency pass [120]. Achalasia, with its main symptom of dysphagia, can lead to malnutrition and chronic pain; its treatment involves endoscopic balloon dilation or surgical myotomy of the lower esophageal sphincter [121]. For alacrimia, artificial tears can be substituted to prevent chronic irritation and inflammation of the cornea [116].

ALADIN is a beta-barrel nucleoporin that interacts with NDC1 [122, 123]. The AlphaFold-predicted structure of an NDC1-ALADIN dimer can be accurately fitted into the Cryo-EM structure of the Xenopus or human NPC [4, 124]. Interestingly, patients with biallelic *NDC1* variants exhibit the *AAAS*-like symptoms alacrima, achalasia, and neurological features but lack signs of adrenal insufficiency [125]. Although ALADIN is expressed throughout the body, the highest levels are found in the adrenal glands, pituitary, and central nervous system. Pituitary dysfunction is uncommon, although isolated cases of growth hormone deficiency have been reported. Most disease-causing variants lead to the mislocalization of ALADIN to the cytoplasm, including a variant originally named p.Gln15Lys, which was later identified to affect splicing (43 C > A) leading to truncated protein that is subjected to degradation [126]. Therefore, ALADIN’s mislocalization away from the NPC appears to be the molecular driver of disease. Although there are three mutation hotspots, “p.Gln15Lys” p.Ser263Pro, and IVS14 + 1G > A, disease-causing variants are scattered throughout the entire protein. Since most of these are not directly at the NDC1-ALADIN structural interface, they likely result in a generally misfolded or unfolded protein that cannot be recruited to NPCs. Interestingly, despite high evolutionary conservation, *Aaas* knockout mice remain viable and do not exhibit the overt features seen in human Tripple A syndrome [127].

It has been suggested that mislocalization of ALADIN leading to a specific failure in nuclear protein import is the primary defect associated with the disease. Specifically, a mutant form of ALADIN, caused by a p.Ile482Ser variant, reduces the nuclear import of DNA ligase 1, aprataxin [128], and ferritin heavy chain (FTH1) [129]. These proteins play roles in DNA damage defense and repair. DNA ligase I and aprataxin are involved in the repair of DNA single-strand breaks. FTH1 associates closely with DNA, preventing damage from oxidative stress, and nuclear FTH1 protects cells against iron and UV-induced oxidative harm. Therefore, the ALADIN-associated import defect increases vulnerability to oxidative stress and may result in cell death. The mechanism by which these changes in nuclear import amplify intracellular levels of reactive oxygen species (ROS) still requires clarification. Increased oxidative stress has been linked to apoptosis and tissue damage in patients with Triple A syndrome. Multiple in vitro studies have shown oxidative stress in cultured adrenal, neuronal, and fibroblast cells with *AAAS* mutations [128, 130, 131]. ALADIN knockdown in a human adrenocortical tumor cell line results in significant impairments in steroid production, particularly in glucocorticoid and androgen pathways, and hinders cellular responses to oxidative stress [132]. This suggests that Triple A syndrome involves defects in both nuclear transport and redox balance. After exposure to chronic oxidative stress, *Aaas* knockout mice exhibited a surprising compensatory response in glutathione metabolism, probably compensating for the loss of ALADIN function [133]. One study proposed that antioxidant therapy could be a promising approach to slow or prevent the progression of Triple A syndrome [131]. In a case report, treatment with N-acetylcysteine in a boy with Triple A syndrome lowered reactive oxygen species levels [134]. However, the long-term effectiveness and potential of N-acetylcysteine in preventing disease-related degeneration still require thorough investigation.

Beyond its role in nuclear import, ALADIN also participates in mitotic spindle assembly and positioning by regulating Aurora A localization at centrosomes [135]. Some of the mitotic phenotypes observed in patient fibroblasts, such as mitotic errors, might therefore be part of the syndrome’s etiology. In murine models, ALADIN deficiency impairs oocyte maturation, spindle positioning, and polar body extrusion [136], which explains the infertility in female *Aaas* KO mice [127]. However, female infertility has not been reported in human patients [137]. Although it has been suggested that, despite the lack of obvious AAAS-like symptoms in *Aaas* knockout mice, these mitotic defects may contribute to the human disease, further evaluation of this hypothesis is needed.

### Nucleoporins in steroid-resistant nephrotic syndrome

While typically one or a few nucleoporins are associated with specific disease phenotypes discussed so far, several structural nucleoporins have been linked to the kidney disease known as steroid-resistant nephrotic syndrome (SRNS). Nephrotic syndrome (NS) describes a diverse group of kidney diseases characterized by the loss of protein in the urine (proteinuria), leading to edema, pleural effusion, and ascites. NS results from damage to the kidney’s glomerular filter, where highly specialized epithelial cells called podocytes form a three-layered filtration barrier that normally retains blood cells and plasma proteins while filtering water and dissolved solutes. At the cellular level, podocytes are the primary site of damage in NS. The NS causes are diverse, including autoimmune diseases, metabolic conditions like diabetes, obesity, hypertension, and inherited disorders. Immune-mediated NS, also known as primary NS, is treated with immunosuppressive therapy, where corticosteroids are the first-line treatment. Second-line treatments are employed for cases that are resistant to steroids, prone to relapse, or have side effects that prevent continued steroid use. These options include calcineurin inhibitors such as Cyclosporin A or Tacrolimus, as well as B-cell targeting agents like Rituximab [138]. In very severe cases of primary NS, plasma exchange is employed to remove disease-causing antibodies. Recently, antibodies targeting the slit diaphragm protein Nephrin have been identified as a common cause of primary NS [139].

Based on the response to steroid treatment, NS can be classified into steroid-sensitive NS (SSNS), which are usually immune-mediated, and steroid-resistant NS, which do not respond to steroid therapy. Additionally, in kidney histology, the two forms differ: SSNS typically shows no obvious abnormalities under conventional light microscopy, with changes only visible through transmission electron microscopy. Therefore, this histological pattern is called “minimal change disease” (MCD). In contrast, SRNS is associated with the histological pattern of focal segmental glomerulosclerosis (FSGS), indicating irreversible scarring of the glomerulus. While MCD is potentially fully reversible in steroid-sensitive patients, it can progress to FSGS in patients who are resistant to treatment. The prognosis for SSNS is generally favorable, while steroid-resistant patients often advance to chronic kidney disease and end-stage renal failure [140].

SRNS accounts for approximately 10–20% of NS cases in children but is more prevalent in adults [141]. More than two-thirds of congenital cases, 30% of childhood-onset, and 10–15% of adult-onset SRNS cases are caused by genetic factors (Orphanet.org). The genetic causes of SRNS are diverse and involve more than 30 genes, some of which are extremely rare [142]. Inherited SRNS is usually fully resistant to treatment, but rare cases may respond partially to calcineurin inhibitors. While immune-mediated NS often recurs after kidney transplantation, disease recurrence is very rare in genetic SRNS cases.

In 2015, genetic variants in *NUP107* [143], *NUP93*, and *NUP205* [144] were first identified as monogenic causes of SNRS. Biallelic variants in *NUP107* caused early-onset SRNS around ages 2–3, progressing to end-stage renal failure by age 10, with no reported extra-renal involvement [143, 145]. Similarly, individuals with biallelic variants in *NUP93* showed progressive SRNS with early onset and also no extrarenal features. In contrast, one individual with a NUP205 mutation exhibited SRNS along with congenital heart disease, including malformations of the aortic valve and root [144]. Later, genetic variants in *NUP85*,* NUP133*, and *NUP160* were also recognized as genetic causes of SRNS. As with other nucleoporin genes, the inheritance mode is strictly recessive, with heterozygous carriers of pathogenic alleles appearing phenotypically normal. For affected individuals, SRNS is the main feature of their disease. However, several patients with *NUP107* and *NUP85* variants display variable extrarenal features, especially microcephaly, intellectual disability, short stature, facial dysmorphism, and growth hormone deficiency [146]. Further studies support the presence of neurological, skeletal, and developmental phenotypes in SRNS patients with *NUP85* variants [147]. Additionally, uterine dysplasia [148] and ovarian insufficiency [149] were reported as extrarenal features in an individual with biallelic *NUP160* variants. Overall, the current data suggest that variants in *NUP85*,* NUP93*,* NUP107*,* NUP133*, and *NUP160* cause SRNS with diverse extrarenal features, including cardiovascular disease, short stature, facial dysmorphism, intellectual disability, and most notably, neurodevelopmental disorders.

Interestingly, all six SRNS-linked nucleoporins are part of the structural backbone of NPCs, with NUP85, NUP107, NUP133, and NUP160 as components of the Y-complex. NUP93 and NUP205 are components of the inner and outer rings. It is thus conceivable that nucleoporin-linked SRNS constitutes a pathology linked specifically to structural nucleoporins. Disease-causing variants in SNRS-linked nucleoporins are summarized in Table 1.

**Table 1:** Inherited disorders linked to nucleoporin mutations

Gene	Clinical disorder (MIM number, if applicable)	Mode of inheritance	Affected organs	Phenotypes and clinical symptomes
AAAS	Triple A syndrome (MIM # 231550)	autosomal recessive	Esophagus, lacrimal glands, adrenal gland	• achalasia (spasms of the esophageal spincter) • alacrima (absence of tear production) • adrenal insufficiency, hypocortisolism, occasionally adrenal crisis • additional rare symptoms: o peripheral neuropathy, muscle wasting motor impairment, muscular speech disorder,o autonomic dysfunction, including abnormal sweating, orthostatic hypotensiono skeletal phenotypes: short stature, scoliosis, palmoplantar hyperkeratosis, skin and dental defects
GLE1	Lethal congenital contracture syndrome (MIM # 253310)	autosomal recessive	Syndromic, multi-organ	• congenital deformities and contractures of multiple joints • loss of voluntary muscle movements, muscle wasting • degeneration of motor neurons in the spinal cord • micrognathia (a small jaw), • incomplete lung development • neonatal death
	Congenital arthrogryposis with anterior horn cell disease (MIM # 611890)	autosomal recessive	Syndromic, multi-organ	• contractures, hypotonia • delayed motor development • respiratory failure • facial dysmorphism • skeletal malformations • microcephaly
NDC1	Neurodevelopmental disorder with achalasia, polyneuropathy, and alacrima (MIM # 621328)	autosomal recessive	Brain, nerves, esophagus, lacrimal glands	• intellectual disability, motor delay • peripheral motor neuropathy • muscular weakness • achalasia (spasms of the esophageal spincter) • alacrima (absence of tear production)
NUP37	Atrial fibrillation, sudden cardiac death (n/a)	autosomal dominant	Heart	• rapid, irregular heart rhythm, leading to palpitations, and potentially impaired cardiac function with shortness of breath, dizziness, and exercise intolerance. A major risk factor for ischemic stroke • sudden cardiac death typically results from fatal ventricular arrythmias
	autosomal-recessive primary microcephaly (MIM # 618179)	autosomal recessive	Brain, skeletal system	• congenital microcephaly, cerebellar hypoplasia • intellectual disability • clinodactyly of the fifth finger
NUP62	infantile bilateral striatal necrosis (MIM # 271930)	autosomal recessive	Brain (basal ganglia)	• progressive neurodegenerative disorder • developmental regression, loss of motor skills • seizures • choreiform hyperkinetic movements • muscular spasms • swallowing difficulties • early lethality
NUP85	Steroid-resistant nephrotic syndrome (MIM # 618176)	autosomal recessive	Kidney	• proteinuria (urinary loss of protein), edema, progressive kidney failure
	Seckel syndrome, or autosomal recessive congenital microcephaly (n/a)	autosomal recessive	Brain, head/face, skeletal system	• microcephaly leading to severe intellectual disability and developmental delays • dysmorphic features • dwarfism • skeletal malformations
NUP88	Fetal akinesia deformation sequence (MIM # 618393)	autosomal recessive	Syndromic, multi-organ	• restriction of fetal movements due to developmental defects in the neuromuscular axis • intrauterine growth retardation • joint contractures • lung hypoplasia • cardiac defects • neonatal lethality or stillbirth
NUP93	Steroid-resistant nephrotic syndrome (MIM # 616892)	autosomal recessive	Kidney	• proteinuria (urinary loss of protein), edema, progressive kidney failure
	Isolated non-progressive congenital ataxia (n/a)	autosomal recessive	Brain (cerebellum)	• neuromotor delay • signs of cerebellar dysfunction (tremor, ataxia, poor coordination, decreased muscular tone)
NUP107	Ovarian dysgenesis (MIM # 618078)	autosomal recessive	Female gonads	• lack of spontaneous puberty, absence of menstruation, uterine hypoplasia, and hypergonadotropic hypogonadism
	Steroid-resistant nephrotic syndrome (MIM # 616730)	autosomal recessive	Kidney	• proteinuria (urinary loss of protein), edema, progressive kidney failure
	Galloway-Mowat syndrome (MIM # 618348)	autosomal recessive	Kidney, brain, head	• microcephaly and other developmental brain defects, leading to intellectual disability and developmental delay • facial dysmorphism • proteinuria (urinary loss of protein), edema, progressive kidney failure
NUP133	Steroid-resistant nephrotic syndrome(MIM # 618177)	autosomal recessive	Kidney	• proteinuria (urinary loss of protein), edema, progressive kidney failure
	Galloway-Mowat syndrome (MIM # 618349)	autosomal recessive	Kidney, brain, head	• microcephaly and other developmental brain defects, leading to intellectual disability and developmental delay • facial dysmorphism • proteinuria (urinary loss of protein), edema, progressive kidney failure
NUP155	Atrial fibrillation (MIM # 615770)	autosomal recessive	Heart	• rapid, irregular heart rhythm, leading to palpitations, and potentially impaired cardiac function with shortness of breath, dizziness, and exercise intolerance. A major risk factor for ischemic stroke
NUP160	Steroid-resistant nephrotic syndrome (MIM # 618178)	autosomal recessive	Kidney	• proteinuria (urinary loss of protein), edema, progressive kidney failure
NUP188	Heterotaxie, congenital heart disease (n/a)	autosomal dominant	Developmental defects including the heart	• defects in embryonic left-right patterning, situs inversus (mirror reversal of organs), developmental heart defects
	Sandestig-Stefanova Syndrome (MIM # 618804)	autosomal recessive	Syndromic developmental defects	• congenital cataracts (impaired vision) • hypotonia (reduced muscular tonicity) • developmental brain defects, intellectual disability, developmental delay • congenital heart disease (impaired cardiac function, impaired oxygenation) • central hypoventilation (breathing impairment) • early lethality
NUP205	Congenital heart disease (n/a)	autosomal recessive	Heart	• impaired cardiac function, reduced fitness, impaired blood oxygenation • defects in left-right patterning
	Steroid-resistant nephrotic syndrome (MIM # 616893)	autosomal recessive	Kidney	• proteinuria (urinary loss of protein), edema, progressive kidney failure
NUP210	Congenital heart disease (n/a)	autosomal recessive	Heart	• congenital heart malformations • defects in left-right patterning
NUP214	Acute febrile encephalopathy, increased susceptibility (MIM # 618426)	autosomal recessive	Brain	• fever • sudden changes in mental status • seizures • in some cases: neuroregression, developmental delay, microcephaly, epilepsy, ataxia, brain atrophy, and early death
NUP358	Acute necrotizing encephalopathy/infection-induced acute encephalopathy, increased susceptibility (MIM # 608033)	autosomal dominant	Brain	• acute neurological disorder triggered by infections • loss of consciousness, seizures, coma • rapidly progressing encephalopathy • Variable outcome: ¼ death, ½ long-term neurological disabilities, and ¼ full recovery

For a specific phenotype, Galloway-Mowat Syndrome (GAMOS), a recessive disease characterized by early-onset NS combined with microcephaly, brain development defects, and severe psychomotor developmental delay [150], a genotype-phenotype correlation has been identified between nucleoporin variants and their clinical presentations. Homozygous splicing variants of *NUP133* cause GAMOS [151], while missense variants, which change only a single amino acid, cause isolated SRNS [146, 152]. Similar patterns are observed for *NUP107*, where a homozygous variant (p.Met101Ile) affecting splicing leads to partial skipping of exon 4 and a subsequent reduction in protein levels, resulting in GAMOS [153]. Conversely, other variants lead to isolated NS [145]. This suggests that, at least for NUP107 and NUP133, variants with a greater impact on the protein tend to cause more severe clinical features. This matches the observation that downregulation of NUP133 in zebrafish results in glomerular abnormalities that mimic NS [154] while a presumably more efficient depletion leads to glomerular defects and microcephaly [151]. For NUP93, however, variants that truncate the protein or impair splicing have not been reported in a biallelic state, suggesting that a complete loss of NUP93 may be incompatible with survival.

Among the SRNS-linked nucleoporins, genetic *NUP93* variants are the most prevalent. Within exon 16, a mutational hotspot, the p.Gly591Val allele likely represents an Eastern European founder allele [155]. No clear pattern for specific regions or domains has been observed in the locations of genetic variants across different nucleoporins. The only exception is that variants in NUP93 do not affect the N-terminal region, which interacts with the NUP62 complex of the central channel. Instead, all variants affect the central or C-terminal regions, where NUP93 interacts with the NPC’s structural scaffold.

The observation that FSGS-linked nucleoporins are all part of the inner or outer ring substructure indicates a functional connection between NPC scaffold malfunction and the development of SRNS. The common phenotype suggests a class effect rather than a role for individual nucleoproteins outside the NPC. Given the fundamental and universal role of NPCs in eukaryotic cells, it remains unclear why nucleoporin variants appear to target particular cell types preferentially. As podocytes and neurons share key features and are both post-mitotic, highly branched, and long-lived cells, this might explain the phenomenon. As podocytes populate the epithelium of kidney glomeruli, these cells are exposed to large fluctuations in osmotic environment, which in turn could impact nuclear envelope tension. As the resulting fluctuations in nuclear envelope tension put stress on the NPC’s structural backbone, SRNS-linked mutations in scaffold nucleoporins may weaken NPC stress resistance and cause NPC dysfunction [156]. However, in this context, it remains unclear why some nucleoporin variants cause Galloway-Mowat Syndrome with additional neuronal phenotypes. In neurons, embedded in a soft-tissue environment of the brain, NPCs are predictably exposed to less mechanical stress. Therefore, multiple molecular disease mechanisms may exist.

Various model systems are used to investigate the molecular mechanisms behind nucleoporin-related kidney disease: In biopsies from patients, distorted nuclei were observed [157], and patient-derived fibroblasts support the idea of abnormal nuclear morphology and a decrease in NPC number when certain nucleoporin variants are present [158]. Additionally, one study found signs of stress in the endoplasmic reticulum, leading to the speculation that processing and trafficking of mutated nucleoporins may be disrupted [157], which could eventually result in reduced mutant nucleoporin levels within NPCs. Cell culture models with genetic nucleoporin inactivation are used for mechanistic studies, revealing several molecular defects, including impaired nuclear import of transcription factors, changes in transcriptional regulation, and nuclear compartmentalization. In particular, loss of NUP93 in podocytes impairs the nuclear import of the transcription factor SMAD4, which regulates BMP-related signaling and may influence podocyte survival [144]. Similarly, NUP205 was identified as an essential factor for the nuclear import of transcription factors YAP/TAZ, which are important mediators of the Hippo-signaling pathway [159]. More than other cell types, podocyte health depends heavily on the presence of YAP/TAZ in the nucleus [160], suggesting that loss of signal networks that maintain podocyte survival may contribute to the disease phenotype. Knockdown of NUP133 not only impaired NPC formation and reduced NPC abundance but also caused distinct changes in the podocytes’ transcriptome [161]. This suggests that imbalances in transcriptional programs are a key driver of molecular pathogenesis. For NUP133, altered transcription is prominently associated with regulators of the actin cytoskeleton, especially RHO-GEF and RHO-GAP, which control RHO-GTPases. Consistent with this, loss of NUP133 [161], as well as NUP85 and NUP107 [146], disrupts cytoskeletal architecture and affects cell spreading and shape. Notably, because of their highly branched structure and exposure to mechanical stress in the glomerular filter, podocytes are particularly dependent on a dynamic and tightly regulated actin cytoskeleton. A mouse model with a podocyte-specific NUP160 knockout recapitulates the human kidney phenotype and shows dysregulation of the small GTPase CDC42, another major regulator of the podocyte’s actin cytoskeleton [162, 163].

Finally, nuclear compartmentalization was found to be disrupted upon NUP93 knockdown, resulting in the diffusion of nuclear proteins into the cytoplasm [164]. Not only might this molecular defect worsen the reduced nuclear abundance of transcription factors caused by trafficking issues, but it could also hinder various other nuclear processes. Generally, loss of nuclear-cytoplasmic compartmentalization is recognized as a major contributor to aging, especially in post-mitotic cells, where long-lived nucleoporins accumulate oxidative damage, and NPCs lose their barrier capability over time [165, 166]. Consistent with the harmful effects of nucleoporin loss, a *Drosophila melanogaster nup93* knockdown model shows reduced cell numbers, disrupted nuclear morphology, and signs of apoptosis [167]. Similarly, in NUP160-deficient flies, the number of nephrocytes, which are analogous to kidney cells, is significantly reduced during development and becomes undetectable in adulthood, indicating a lethal effect of NUP160 depletion [168]. These flies also show severe disruption in nuclear morphology and NPC assembly, suggesting that the absence of structural nucleoporins significantly impairs normal NPC function.

Defective NUPs are associated not only with aging but also with development and stem cell differentiation, where, for example, the absence of NUP133 disrupts neuronal differentiation in mice [169]. NUP133-deficient stem cells stay in a pluripotent state but fail to differentiate into the neuronal lineage. Recently, it was shown that this occurs due to NPC disintegration, leading to nuclear envelope breakdown during mechanical stress in development [170]. Loss of NUP85, NUP107, and NUP133 impairs glomerular development in Xenopus tropicalis larvae [146]; morpholino knockdown of NUP107 in zebrafish results in dysplastic glomerular structures [143]; and CRISPR-Cas9-mediated knockdown of NUP85 and NUP107 causes developmental defects in zebrafish [146], suggesting that SRNS-related nucleoporins may also have roles in development. A genetic mouse model of *NUP93* deletion in mature podocytes revealed that, in the early disease stages, NUP93 loss reduced the intranuclear levels of specific podocyte transcription factors, such as Wilms tumor 1 (WT1), LIM Homeobox Transcription Factor 1 Beta (LMX1B), and V-maf musculoaponeurotic fibrosarcoma oncogene homolog B (MAFB) [171]. The finding that podocyte dedifferentiation caused by NUP93 loss is a key factor in nucleoporin-related inherited kidney disease was further confirmed by experiments showing that transient siRNA-mediated NUP93 knockdown in cell cultures also lowers transcription factor levels. This decline may stem from reduced nuclear entry or enhanced passive diffusion of transcription factors out of the nucleus, potentially due to the increased leakiness of NPCs lacking NUP93. Moreover, NUP93 depletion decreased NPC density in podocyte nuclei. In advanced disease stages, NUP93 deficiency activated the DNA damage response, signaling genomic injury, and resulted in podocyte apoptosis. Genetic inactivation of NUP93 early during kidney development causes embryonic lethality in mice [171].

In summary, several molecular mechanisms have been proposed to explain the link between nucleoporin variants and SRNS. Supplemental Table 1 provides a list of all nucleoporin variants detected in SRNS patients to data. Gaining a better understanding of this pathogenic connection will aid in identifying new molecular targets for therapy, such as the nuclear export inhibitor selinexor, which shows promising results in a rat FSGS model [164]. To date, no targeted treatment exists for inherited SRNS. Immunosuppressive therapy, the standard care for primary, immune-mediated NS, generally does not have a consistent positive effect on inherited SRNS. Therefore, current management depends on supportive measures, including blood pressure control and reducing proteinuria through inhibition of the Renin-Angiotensin-Aldosterone system and SGLT2 (sodium/glucose cotransporter 2) inhibitors. Additionally, dietary adjustments such as salt reduction and decreased protein intake are used, and nephrotoxic drugs are avoided when possible. As the disease progresses, edema becomes more severe and can be managed with diuretics. Severe urinary protein loss may lead to endocrine disorders like hypothyroidism, elevate the risk of infections due to loss of protective antibodies, and cause coagulation imbalances that increase the risk of thrombosis and pulmonary embolism. Furthermore, ongoing scarring of the kidney’s glomerular filter leads to progressive chronic kidney disease, which causes electrolyte imbalances, volume overload, anemia, bone disease, and eventually uremia, i.e., high levels of urea in the blood. This condition requires kidney replacement therapy or transplantation for survival. Unlike immune-mediated NS, inherited SRNS rarely recurs after a kidney transplant, often making transplantation curative. Interestingly, some NUP93 genetic variants exhibit atypical disease mechanisms that differ from the usual pattern of inherited SRNS (Table 1). Notably, certain patients, especially those with the p.Gly591Val variant of NUP93, show partial improvement with intensified immunosuppressive therapy using Ciclosporin A [172]. Moreover, disease recurrence after transplantation has been documented in two cases: one involving a compound heterozygous p.Gly591Val/p.Leu639Pro variant of *NUP93* [173] and another with the p.Lys637Glu variant of *NUP93* [167]. Both cases responded to plasma exchange, and one also responded to B–cell–depleting therapy with Rituximab, indicating at least a partially immune-mediated mechanism. Most patients with nucleoporin-associated SRNS, however, rapidly progress to end-stage kidney disease (ESKD) despite optimal medical management. As a result, existing treatments may slow but not halt the progression of inherited FSGS, emphasizing the urgent need for new therapeutic options [174].

### Nucleoporins in neurological disorders

#### Nucleoporins of the inner and outer rings in developmental brain disorders

Beyond the renal-neurological phenotypes described in the previous chapter for certain genetic variants in the Y-complex nucleoporins NUP107 and NUP133, nucleoporins have been associated with various other neurological disorders. For NUP85, which is also an SRNS-associated nucleoporin and part of the NUP107-NUP160 complex, the situation is even more complicated because, depending on the specific allele, genetic variants in *NUP85* are linked to either kidney disease or to autosomal recessive congenital microcephaly and Seckel syndrome spectrum disorders without kidney features [175]. Seckel syndrome is characterized by microcephaly leading to severe intellectual disability and developmental delays, along with distinct dysmorphic features, dwarfism, and skeletal malformations. Due to the rarity of *NUP85* variants, the exact relationship between genotype and phenotype, and the molecular mechanisms underlying the different phenotypic outcomes, remains unclear. NUP37, another subunit of the NUP107-NUP160 complex, was found to have a homozygous genetic variant in a family with three children exhibiting autosomal-recessive primary microcephaly (MIM #618179). In this case, a homozygous nonsense variant caused the absence of the NUP37 protein and defects in nuclear structure [146].

Among components of the NUP93-NUP205 complex, NUP93 was most notably linked to the isolated kidney phenotype of SNRS. However, in two siblings with isolated non-progressive congenital ataxia, two rare compound heterozygous variants in *NUP93* were identified, indicating a possible connection between certain *NUP93* variants and developmental brain defects [176]. In this condition, cognitive development remains normal, while neuromotor development is delayed, and characteristic signs of cerebellar dysfunction, such as ataxia, tremor, poor coordination, decreased muscular tone, as abnormal eye movements are observed. For NUP188, the NUP205 orthologue and an interaction partner of NUP93, biallelic nonsense variants lead to a severe, multiorgan, syndromic condition called Sandestig-Stefanova Syndrome (MIM #618804) [70, 177]. Major features include severe developmental brain defects, especially microcephaly, a hypoplastic corpus callosum, white-matter lesions, and delayed myelination, resulting in profound developmental delays, failure to thrive, and death within the first year of life due to central respiratory failure. Additionally, affected children often have congenital heart defects, skeletal malformations, and dysmorphic facial features, suggesting that biallelic loss-of-function in NUP188 causes significant defects across multiple developing organ systems, making NUP188 another example of null mutations in nucleoporins linked to developmental CNS abnormalities. In line with this finding, *nup188* loss-of-function variants in *Drosophila melanogaster* lead to early lethality and severe neurodevelopmental defects, particularly affecting dendrite and neural circuit formation [70].

#### Nucleoporins in infection-induced encephalopathies

Biallelic genetic variants in *NUP214* are associated with acute febrile encephalopathy (AFE), a medical emergency characterized by fever, sudden changes in mental status (such as confusion or coma), and/or seizures, indicating brain damage. It can result from various underlying conditions, including bacterial, viral, or parasitic infections of the central nervous system, as well as non-infectious causes like metabolic disorders, sepsis, or autoimmune diseases. One rare subtype, IIAE9, is a neurodevelopmental disorder caused by genetic variants in *NUP214*, which makes individuals susceptible to infection-induced encephalopathy (MIM# 618426). This disorder usually appears in infants or young children and may cause neuroregression, developmental delays, microcephaly, epilepsy, ataxia, brain atrophy, and early death [178, 179], but patients might also recover. Most affected individuals carry either homozygous (p.Arg38Cys, p.Asp154Gly) or compound heterozygous missense or frameshift variants in *NUP214*, such as p.Pro387Ser with p.Pro525Leufs∗6 or p.Arg38Cys with p.Ile310Thr. While most patients are young children, a 20-year-old male patient with episodic ataxia, seizures, and encephalopathy triggered by febrile illness has been found to have an in-frame deletion (p.Leu68del), suggesting that the condition can also present later in life in the presence of milder genetic variants [180].

NUP214, also known as CAN, is found on the cytoplasmic side of NPCs [181] and is part of a trimeric complex with NUP88 and NUP62 [7, 101]. It plays a crucial role in CRM1-mediated nuclear export [21, 104] and interacts with the helicase DDX19 (also known as DBP5) during mRNA export [182]. Consequently, cells lacking NUP214 show impaired nucleocytoplasmic transport, an accumulation of polyadenylated RNA in the nucleus but also a cell cycle arrest [183]. In mice, knocking out *Nup214* is embryonic lethal [183].

Functional analyses of IIAE9 patient fibroblasts showed reduced NUP214/NUP88 levels, mislocalized NUP214, and impaired nucleocytoplasmic transport, causing accumulation of mRNA complexes, described as “plugged nuclear pore complexes” [178, 179]. This indicates that defects in nucleo-cytoplasmic transport are key molecular mechanisms in disease development [179]. When exposed to stress, patient-derived fibroblasts show a compromised stress response and increased apoptosis. However, co-expressing wild-type NUP214 and NUP88 partially rescues this apoptosis phenotype [179]. An in-frame deletion in *NUP214* was described in a male with infection-induced encephalopathy, who survived into adulthood with recurring seizures and ataxia, and brain imaging showed progressive cerebellar degeneration [180]. The differing ages of onset and severity across these reports suggest a complex genotype-phenotype relationship that remains poorly understood.

Acute necrotizing encephalopathy (ANE, MIM #608033) is a rare neurological disorder mainly caused by viral infections such as influenza and parainfluenza, primarily affecting young children aged 1–4 years but also occurring in adults. It manifests with symptoms like loss of consciousness, seizures, coma, and rapidly progressing encephalopathy. Most ANE cases are sporadic and non-familial; however, familial cases tend to be recurrent [184]. About 75% of these familial cases are linked to heterozygous missense variants in the gene *RANBP2* and are classified as ANE1 [185], autosomal dominant acute necrotizing encephalopathy (ADANE), or infection-induced acute encephalopathy 3 (IIAE3). The penetrance is approximately 50%. To date, 96 ANE1 patients have been reported worldwide, mostly from North America and Europe. Increased levels of interleukin 6 and TNF-alpha have been observed in the serum and cerebrospinal fluid of ANE patients, leading to the suggestion of a “cytokine storm’ as part of the disease’s underlying mechanism. Among reported outcomes for ANE1 patients with available data, 25.4% died, 52.1% have long-term neurological disabilities, and 22.5% fully recovered. Most missense variants occur in the N-terminal part of *RANBP2* (p.Asp43Val, p.Leu450Phe, p.Thr585Met, p.Thr653Ile, p.Ile656Val, p.Trp681Cys), with p.Thr585Met being the predominant variant, accounting for over 70% of all cases. Variants such as p.Lys1121Asn, p.Lys1665Glu, and p.Pro1750Arg are located more toward the C-terminal end [184, 185].

RANBP2, also known as NUP358, is the largest nucleoporin and a key component of the cytoplasmic filaments of the NPC [3]. It is an extended, multidomain protein composed of an N-terminal 75 kDa S-shaped α-helical solenoid domain, which is important for anchoring RANBP2 to the NPC, followed by a coiled-coil oligomerization element, numerous Ran-interacting domains, an E3 ligase domain, and a C-terminal prolyl-isomerase domain [7, 186, 187]. The E3-ligase domain attaches SUMO to protein substrates and interacts with Ubiquitin Conjugating Enzyme 9 (UBC9) and SUMO-modified Ran-GTPase activating protein (RANGAP1). The formation of the RANBP2/SUMO-RANGAP1/UBC9 complex at NPC cytoplasmic filaments is crucial for its SUMO E3-ligase activity [188–190]. Beyond SUMOylation, RANBP2 is involved in various cellular processes such as nucleocytoplasmic transport, mRNA metabolism, microRNA-induced silencing, and microtubule-kinetochore attachment during mitosis [7, 191–194].

Five copies of RANBP2 are present at each of the eight spokes of the NPC, totaling 40 copies per NPC [7]. The N-terminal domain of each RANBP2 monomer consists of three distinct alpha-helical solenoids, forming a unique overall S-shaped structure and containing several mutation sites linked to ANE1. Among these, p.Trp681Cys, p.Tyr653Ile, and p.Ile656Val do not disrupt the fold or affect the normal cellular function of RANBP2 as tested, but they do reduce the thermostability of this domain. It has been suggested that a fever-inducing trigger during viral infection damages RANBP2 stability and likely its localization to the NPC, which could explain the sudden onset of symptoms during infection [7]. However, this domain is also required for the interaction with microtubules [195], and it remains uncertain whether the other mutations outside this solenoid domain similarly impact RANBP2 stability.

How does RANBP2 relate to the cytokine storm in ANE? It has been reported that RANBP2 represses the translation of IL-6. In this context, RANBP2 SUMOylates Argonaut proteins, which stabilizes the associated miRNA-induced silencing complex containing IL-6 mRNA and let-7 miRNA. This stabilization ultimately leads to the repression of IL-6 translation [196]. It is thus possible that the proposed destabilization of RANBP2 ANE1 variants upon viral infection partially releases this translational repression. Interestingly, in tissue culture models ANE1-linked RANBP2-variants still SUMOylate argonaut proteins [196], consistent with the idea that it requires a trigger during viral infection. Genetic variants in RANBP2 increase the risk of developing ANE, but they are not sufficient on their own to fully manifest the phenotype. It is still unclear whether additional genetic or environmental factors are necessary for this condition to occur.

#### Nucleoporins in neurodegenerative diseases

Unlike the neurodevelopmental phenotypes mentioned earlier, where brain defects are present at birth, genetic variants in *NUP62* lead to a progressive neurodegenerative disorder, specifically infantile bilateral striatal necrosis (MIM #271930), where the basal ganglia gradually decline [197]. Affected children show developmental regression within the first year, along with a continuous loss of neuromotor skills, seizures, choreiform hyperkinetic movements (i.e., involuntary, irregular, and unpredictable motions of the limbs, face, neck, and trunk), muscle spasms, and swallowing difficulties. The disease course is fatal.

NUP62 is an FG-nucleoporin that, with its partners NUP58 and NUP54, forms most of the gel-like matrix in the central NPC channel. It also belongs to a different NPC subcomplex interacting with NUP214, NUP98, NUP88, NUP42, RAE1, and GLE1 at the base of the cytoplasmic filaments. Since a similar phenotype involving early-onset dystonia with degeneration of striatal neurons was later described for NUP54 [198], it is likely that the central NPC channel’s function is affected in infantile bilateral striatal necrosis. Interestingly, the variants are found as clusters in the C-terminal region of NUP54, which mediates interaction with NUP62, suggesting another “class phenotype” - similar to what is seen with Y-complex nucleoporins and SRNS - where genetic variants in different NPC subunits result in similar clinical features.

Dysfunction of NPCs and altered nuclear transport have also been linked to major neurodegenerative diseases such as amyotrophic lateral sclerosis (ALS), frontotemporal dementia, Alzheimer’s disease, and Huntington’s disease (reviewed in [199]). This was first described for ALS (MIM #105400), a progressive neurodegenerative disease mostly caused by a GGGGCC (G_4_C_2_) hexanucleotide repeat expansion (HRE) in the *C9ORF72* gene. ALS is the most common adult-onset motor neuron disease, leading to death within a few years of onset due to progressive paralysis [200]. It is caused by the simultaneous degeneration of upper motor neurons in the motor cortex and lower motor neurons in the brainstem and spinal cord, leading to muscle weakness, paralysis, and eventual respiratory failure. A subset of ALS patients also suffers from frontotemporal dementia (FTD), which results from a similar pathogenesis in a different neuronal cell type [201]. Key clinical features of FTD include changes in behavior and personality, emotional dysregulation, repetitive behaviors, and aphasia. About 50% of ALS patients have mild cognitive deficits, while approximately 10% of patients show significant signs of both FTD and ALS. ALS can be classified as either familial or sporadic, with variants in four genes (*C9ORF72*, *SOD1*, *TARDBP*, and *FUS*) accounting for most familial cases. Variants in three genes (*MAPT*, *GRN*, and *C9ORF72*) account for most cases of familial FTD. However, both ALS and FTD are linked to an increasingly expanding list of additional genes [202].

In 2015, two elegant studies observed that nucleocytoplasmic transport was impaired in ALS-derived induced pluripotent stem cells, suggesting it could be targeted therapeutically [203, 204]. One study found impaired nuclear protein import and showed that enhancing nuclear import while suppressing nuclear export improved neurodegenerative phenotypes [203]. The other study observed defects in RNA export, leading to RNA retention in the nucleus [204]. Nuclear transport receptors and mediators of Ran-dependent nuclear transport were identified as modifying factors in ALS [205]. Whether toxic cytoplasmic protein aggregates in ALS patients are a result of, or a cause of, impaired nuclear transport, or represent an independent pathogenic mechanism, remains to be determined [199]. A subsequent study suggested a primary defect in NPC composition, characterized by a reduced abundance of core structural components within NPCs of ALS patient cell lines. NPC disruption was proposed to secondarily lead to defects in nuclear transport, misdistribution of RanGTP, and nucleo-cytoplasmic mislocalization or accumulation of protein aggregates [206, 207]. The transmembrane nucleoporin POM121 was identified as an integral regulator of proper NPC composition that can rescue or mimic the ALS phenotype of NPC injury [206].

Genetic variations in the nucleoporins *GLE1* and *NUP50* are also associated with ALS. Disease-specific *GLE1* variants are found in a small subset of ALS patients, including both familial and sporadic cases [208]. The study identified two detrimental variants, a splice site variant and a nonsense variant, as well as one missense variant, in *GLE1*. A transcriptome-wide association study on a dataset of thousands of ALS patients [209] identified NUP50 as a disease-related transcript. Both coding and non-coding variants in *NUP50* were described as potential ALS risk variants, using samples from 41 patients. Notably, a prior study using a *Drosophila* model showed that reducing NUP50 levels increased the toxicity of ALS-associated C9ORF72-like repeat expansions [204].

GLE1, discussed already above in the context of LCCS1 and CAAHD, is a DEAD-box RNA helicase that plays a crucial role in mRNA export and is also involved in efficient translation initiation and termination [94]. In humans, two isoforms of GLE1 (GLE1A and GLE1B) are expressed. GLE1A is distributed throughout the nucleus and cytoplasm, while the longer GLE1B isoform is mainly found on the cytoplasmic face of the NPC through interactions with NUP42 and NUP155 [54, 55]. In HeLa cells, both harmful mutants caused a loss of GLE1 at NPCs, while the mutant protein was mislocalized to the cytoplasm. Thus, it was proposed that increased cytoplasmic activity of GLE1 contributes to the molecular pathogenesis and that the mutant protein, due its ability to mimic GLE1A and GLE1B activity, disrupts the natural balance between the two isoforms and the control of GLE1 activity [210]. As mentioned earlier, morpholino-mediated knockdowns in zebrafish led to severe developmental defects, including motor neuron arborization abnormalities and embryonic lethality [95], which were rescued by expressing wild-type GLE1B, but not the two mutant versions [208]. As previously noted, different *GLE1* variants can cause Leigh syndrome with cerebellar hypoplasia (LCCS1) and congenital ataxia with axonal neuropathy and hypomyelination (CAAHD), and it remains an interesting question what functional and cellular differences of GLE1 produce these distinct disease outcomes.

NUP50 is localized to the nuclear side of the NPC and has an auxiliary role in nuclear transport [211, 212], but it is also associated with gene expression regulation and cell cycle control [213, 214]. The *Nup50* knockout mouse is embryonically lethal, with mice dying in utero due to severe neural tube defects [214]. In *Drosophila* and zebrafish, downregulation of NUP50 results in motor neuron defects [209]. However, it remains unclear which function of NUP50 is affected in different NUP50 variants, especially since the mutations in the coding region are scattered throughout the protein, including its importin α-, RCC1-, importin β-, and Ran-binding sites, as well as the NPC-targeting site. Cells from an ALS patient carrying one of the potential ALS risk variants exhibited reduced NUP50 protein expression [209]; however, it is unknown whether this reduction occurs in all other variants.

Huntington disease (HD, MIM #143100), a neurodegenerative disorder that causes involuntary, hyperkinetic movements, mood disorders, and dementia, results from a trinucleotide repeat expansion leading to the buildup of toxic protein aggregates within neuronal cells. The disease has a chronic, progressive course for which there is currently no cure and targeted treatment.

Like in ALS, these protein aggregates have been found to disrupt global nucleocytoplasmic transport, as measured by a nuclear transport reporter mislocalization, and to cause cytoplasmic aggregation and mislocalization of NPC components [215]. Mutant Huntingtin, the protein mutated in Huntington disease, sequesters GLE1 and RANGAP1, both essential for nucleo-cytoplasmic transport, thereby causing defects in nuclear mRNA export and leading to nuclear RNA accumulation [216].

Alzheimer’s disease (AD; MIM #104300), one of the most common forms of dementia that mostly affects older adults, results from abnormal accumulation of misfolded amyloid beta peptides and hyperphosphorylated tau proteins in the brain, which leads to neurodegeneration.

As with other neurodegenerative diseases, defects in nucleocytoplasmic transport have also been described in AD. Tau proteins were found to sequester NPC components, thus disrupting NPC integrity and impairing nuclear import and export by mislocalizing Ran [217]. Neurons with a buildup of neurofibrillary aggregates in AD were found to have misshaped nuclei and cytoplasmic aggregates of the nuclear transport factor 2 (NTF2), indicating a mislocalization of nuclear transport receptors. Additionally, AD nuclei were found to have defects in the nucleocytoplasmic barrier, as demonstrated by leakiness in a dextran exclusion assay [217]. In summary, different neurodegenerative diseases share common mechanisms, including NPC dysfunction, nucleoporin mislocalization, and defective nucleocytoplasmic transport.

Interestingly, it was observed that pathogenic features seen in neurodegenerative diseases also occur during aging (reviewed in [218]). Specifically, NPCs lose their effective barrier function and become leaky, allowing passive diffusion of nuclear and cytoplasmic proteins in aging neurons [165]. Furthermore, aging fibroblasts show a decrease in nuclear transport proteins like RANBP1, resulting in reduced nuclear import [219]. Aging yeast cells accumulate misassembled NPCs [220]. Post-mitotic cells like neurons and glomerular podocytes are more vulnerable to NPC damage during aging compared to rapidly dividing cells because NPC turnover is very limited in non-dividing cells [165]. While NPC composition stays stable in young cells despite the lack of turnover, aging cells exhibit a decrease in some nucleoporins and alterations in NPC makeup [221], which may drive NPC defects.

## Concluding remarks

In summary, nucleoporins are involved in various monogenic diseases. While some, such as Triple-A syndrome, present systemically, others show phenotypes predominantly in certain cell types, tissues, and organelles. This specificity is surprising given the crucial role of NPCs in all nucleated cells for nuclear-cytoplasmic compartmentalization and transport. It is generally assumed that NPCs in different cell types have a mostly identical composition of nucleoporins, with few notable exceptions, such as NUP210. Whether subtle differences in the relative stoichiometry of NPCs across cell types exist, and if these differences influence susceptibility, remains a key question. The observed overexpression of certain nucleoporins in some disease entities, e.g., NUP188 in CHD, could point in this direction.

For some diseases, it may be hypothesized that a moonlighting function of the involved nucleoporin outside of NPC-related roles could cause the disease; this is plausible, for example, in nucleoporin dysfunction linked to defects in left-right patterning. In contrast, for other diseases like SRNS, given the number of nucleoporins involved, NPC dysfunction appears highly likely.

Currently, treatments for the discussed monogenic disorders are mainly symptom-based and only slow disease progression, rather than providing a cure. Furthermore, current treatments are quite non-specific, bearing the risk of significant side effects. Therefore, there is an urgent need for a deeper molecular understanding of the defects caused by mutations in nucleoporin-encoding genes in relevant cell types. This knowledge could help identify dysregulated pathways as targets for molecular therapies, ultimately leading to the development of more effective, targeted therapies for these diseases.

## Supplementary Information

Below is the link to the electronic supplementary material.

Supplementary Material 1

## Data Availability

Not applicable.
