# Supplementary material for: Pathologies at the gateway: exploring the link between nucleoporins and inherited diseases
Source: Cell Mol Life Sci. 2026 Apr 29;83(1):202. doi: 10.1007/s00018-026-06220-2 (PMC13137949; doi:10.1007/s00018-026-06220-2)
Supplement: Supplementary file 1 — Supplementary Material 1 [file 18_2026_6220_MOESM1_ESM.docx]

**Table 1. Genetic variants in nucleoporins linked to nephrotic syndrome**

| **Gene** | **Nucleotide change** | **Amino acid change** | **Exon (Zygosity)** | **Reported individuals** | **Kidney phenotype**  **(age of onset, kidney biopsy)** | **Treatment** | **End-stage kidney disease (ESKD)** | **Kidney transplantation** | **Extrarenal Manifestations** | **Reference** |
| --- | --- | --- | --- | --- | --- | --- | --- | --- | --- | --- |
| ***NUP93*** |  |  |  |  |  |  |  |  |  |  |
|  | **c.554A>G**  **c.2017C>T** | **p. Tyr185Cys**  **p.Arg673Trp** | **6 (het)**  **18 (het)** | **2** | **SRNS (1 yrs)** |  | **ESKD (1 yrs)** | **kidney transplantation (1 yrs)** |  | **Pei et al,**  **Pediatr Res., 2025.** |
|  | **c.565-2A>G**  **c.2137-18G>A** | **skipping of exon 7**  **partial skipping of exon 20** | **intron 7 (het)**  **20 (het)** | **1** | **SRNS (25 yrs),**  **FSGS on biopsy** | **Decision against CNI** | **ESKD (26 yrs)** | **kidney transplantation**  **(no disease recurrence)** | **bilateral optic nerve atrophy, bicuspid aortic valve, osteochondritis dissecans** | **Scheen et al,**  **Kidney Med., 2025.** |
|  | **c.575A>G**  **c.1605C>G** | **p. Tyr192Cys**  **p. Tyr535*** | **7 (het)**  **14 (het)** | **1** | **SRNS (5 yrs)** |  | **ESKD (5yrs)** | **kidney transplantation**  **(no disease recurrence)** | **developmental delay, autism features, dilated cardiomyopathy** | **Sandokji et al,**  **BMC Nephrol., 2019.** |
|  | **c.727A>T**  **c.2137-18G>A** | **p.Lys243***  **skipping of exon 20** | **8 (het)**  **intron 19 (het)** | **1** | **SRNS (2 yrs),**  **FSGS on biopsy** |  |  |  | **optic nerve atrophy** | **Rossanti et al,**  **J Hum Genet., 2019.** |
|  | **c.1162C>T**  **c.2326C>T** | **p.Arg388Trp**  **p.Arg776** | **11 (het)**  **21 (het)** | **1** | **SRNS (3 years),**  **FSGS on biopsy** | **resistant to CNI and Rituximab** | **ESKD (12 yrs)** |  | **mild cardiomyopathy** | **Wasilewska et al,**  **J Clin Med, 2023.** |
|  | **c.1162C>T**  **c.1772G>T** | **p.Arg388Trp**  **p.Gly591Val** | **11 (het)**  **16 (het)** | **1** | **SRNS (6 yrs),**  **FSGS on biopsy** |  | **ESKD (6 yrs)** | **kidney transplantation** |  | **Braun et al,**  **Nat. Genet., 2015.** |
|  | **c.1326delG**  **c.1772G>T** | **p.Lys442Asnfs*14**  **p.Gly591Val** | **12 (het)**  **16 (het)** | **1** | **SRNS (3 yrs),**  **FSGS on biopsy** | **partial response to CNI** | **ESKD (6 yrs)** | **kidney transplantation** |  | **Braun et al,**  **Nat. Genet., 2015.** |
|  | **c.1274A>T** | **p.Asp524Val** | **Hom** | **1** | **SRNS (2 mo),**  **FSGS on biopsy** | **resistant to CNI** | **ESKD (2 yrs)** |  |  | **Tseng et al,**  **J Formos Med Assoc., 2024.** |
|  | **c.1298delA**  **c.1772G>T** | **p.Asp443Alafs*23**  **p.Gly591Val** | **12 (het)**  **16 (het)** | **1** | **SRNS (4 yrs),**  **FSGS on biopsy** | **resistant to CNI** | **ESKD (5 yrs)** | **kidney transplantation**  **(no disease recurrence)** |  | **Bezdíčka et al.**  **Pediatr Nephrol., 2018.** |
|  | **c.1319T>C** | **p.Phe440Ser** | **12 (hom)** | **2** | **SRNS (4 yrs),**  **Severe fibrosis on renal biopsy** |  | **ESKD (4 yrs)** |  |  | **Riyami et al,**  **Mol Genet Genomic Med., 2023.** |
|  | **c.1423G>A** | **p.Ala475Thr** | **13 (hom)** | **2** | **SRNS (1 yr),**  **FSGS on biopsy (collapsing variant in 1 sibling)** |  | **ESKD (2 yrs)** | **kidney transplantation**  **(no disease recurrence)** |  | **Bierzynska et al,**  **Pediatr Nephrol., 2022.** |
|  | **c.1463A>G** | **p.His488Arg** | **13 (hom)** | **1** | **SRNS (2 yrs),**  **Diffuse mesangial sclerosis on biopsy** |  | **ESKD** |  |  | **Dhanorkar et al,**  **Indian J Pediatr, 2024.** |
|  | **c.1472A>T**  **c.1537+1G>A** | **p.His491Arg** | **13 (het)**  **intron 13 (het)** | **1** | **SRNS (1 yrs),**  **FSGS on biopsy** | **resistant to CNI** | **ESKD (1 yrs)** | **kidney transplantation (1 yrs)** | **left atrial and ventricular dilatation, mitral regurgitation, short stature** | **Pei et al,**  **Pediatr Res., 2025.** |
|  | **c.1473T> G**  **c.1538-6A>G** | **p.His491Gln** | **13 (het)**  **intron 13 (het)** | **1** | **FSGS (8 yrs),**  **FSGS on biopsy** |  | **ESKD (9 yrs)** | **kidney transplantation**  **(no disease recurrence)** | **autism spectrum disorder** | **Bierzynska et al,**  **Pediatr Nephrol., 2022.** |
|  | **c.1537+1G>A**  **c.1772G>T** | **skipping of exon 13, in-frame deletion**  **p.Gly591Val** | **intron 13 (het)**  **16 (het)** | **1** | **SRNS (3 yrs),**  **FSGS on biopsy.** |  | **ESKD (4 yrs)** | **kidney transplantation (7 yrs)** | **Marcus-Gunn-**  **Syndrome (unilateral congenital ptosis, elevation of ptotic eyelid upon jaw motion)** | **Braun et al,**  **Nat. Genet., 2015.** |
|  | **c.1537+1G>A**  **c.1772G>T** | **skipping of exon 13**  **p.Gly591Val** | **intron 13 (het)**  **16 (het)** | **1** | **SRNS (3 yrs),**  **MCD on biopsy** |  | **ESKD (3 yrs)** | **kidney transplantation**  **(no disease recurrence)** |  | **Bezdíčka et al.**  **Pediatr Nephrol., 2018.** |
|  | **c.1573C>T**  **c.1886A>G** | **p.Arg525Trp**  **p.Tyr629Cys** | **14 (het)**  **17 (het)** | **1** | **SRNS (4 yrs),**  **FSGS on biopsy (5 yrs)** | **No response to PLEX** | **ESKD (6 yrs)** | **kidney transplantation (15 yrs, no disease recurrence)** | **rheumatoid arthritis** | **Hashimoto et al,**  **Kidney Int Rep., 2019.** |
|  | **c.1604 A > C**  **c.1655 A > G** | **p.Tyr535Ser**  **p.Tyr552Cys** | **14 (het)**  **14 (het)** | **1** | **SRNS (1 yr),**  **FSGS on biopsy** | **resistant to CNI** | **ESKD (2 yr)** |  |  | **Yang et al,**  **Pediatr Nephrol, 2025.** |
|  | **c.1655A>G**  **c.1732C>T** | **p.Tyr552Cys**  **p. Arg578*** | **14 (het)**  **15 (het)** | **1** | **SRNS (1 yr)** |  | **ESKD (1 yr)** |  |  | **Zhao et al,  Medicine (Baltimore), 2021.** |
|  | **c.1604A>C**  **c.1655A>G** | **p.Tyr535Ser**  **p.Tyr552Cys** | **14 (het)**  **14 (het)** | **1** | **SRNS (1 yr),**  **FSGS on biopsy** | **resistant to CNI** | **ESKD (1.5 yrs)** |  |  | **Han et al,**  **Ital J Pediatr., 2024.** |
|  | **c.1772G>T** | **p.Gly591Val** | **16 (hom)** | **2** | **SRNS (3, 6 yrs)** |  |  |  |  | **Braun et al,  Nat. Genet., 2015.** |
|  | **c.1772G>T** | **p.Gly591Val** | **16 (hom)** | **1** | **SRNS (6 yrs),**  **FSGS on biopsy** | **response to CNI (remission)** |  |  |  | **Kuran et al,  Cent Eur J Immunol., 2023.** |
|  | **c.1772G>T** | **p.Gly591Val** | **16 (hom)** | **1** | **SRNS (7 yrs),**  **FSGS on biopsy** | **partial response to CNI** |  |  |  | **Bezdíčka et al.  Pediatr Nephrol., 2018.** |
|  | **c.1772G>T**  **c.1916 T>C** | **p.Gly591Val**  **p.Leu639Pro** | **16 (het)**  **18 (het)** | **1** | **SRNS (2yrs),**  **FSGS on biopsy** | **resistant to CNI** | **ESKD (3 yrs)** | **kidney transplantation (recurrence of disease, dependent on PLEX, sustained remission after Rituximab)** |  | **Bezdíčka et al.  Pediatr Nephrol., 2018**  **Seeman & Vondrak, Transplant Proc., 2018.** |
|  | **c.1772G>T**  **c.2084T>C** | **p.Gly591Val**  **p.Leu695Ser** | **16 (het)**  **19 (het)** | **2** | **SRNS (2, 5 yrs),**  **FSGS (collapsing variant) on biopsy** | **resistant to CNI** | **ESKD (6, 10 yrs)** | **kidney transplantation (7, 10 yrs; no disease recurrence).** |  | **Cason et al,  Front Pediatr., 2022.** |
|  | **c.1886A>G** | **p.Tyr629Cys** | **17 (hom)** | **2** | **SRNS (1 yr)** |  |  |  |  | **Braun et al,**  **Nat. Genet., 2015.** |
|  | **c.1909A>G** | **p.Lys637Glu** | **18 (hom)** | **1** | **SRNS (2 yrs)** |  | **ESKD (3 yrs)** | **kidney transplantation (recurrence of disease, responsive to PLEX)** |  | **Bierzynska et al,  Pediatr Nephrol., 2022.** |
|  | **c.2017C>T**  **c.2258T>A** | **p. Arg673Trp**  **p. Met753Lys** | **18 (het)**  **21 (het)** | **2** | **SRNS (1 yrs),**  **FSGS on biopsy** |  | **ESKD (1 yrs)** | **kidney transplantation** |  | **Pei et al,**  **Pediatr Res., 2025.** |
|  | **c.2084T>C**  **c.2267T>C** | **p.Leu695Ser**  **p.Leu756Ser** | **19 (het)**  **21 (het)** | **1** | **SRNS (6 yrs),**  **FSGS on biopsy** |  | **ESKD (6 yrs)** | **kidney transplantation**  **(no disease recurrence)** |  | **Bierzynska et al,  Pediatr Nephrol., 2022.**  **Bierzynska et al,  Kidney Int., 2017.** |
|  | **c.2141T>C** | **p. Ile714Thr** | **20 (hom)** | **1** | **SRNS (4 yrs),**  **FSGS on biopsy** |  | **ESKD (5 yrs)** | **kidney transplantation**  **(no disease recurrence)** |  | **Acharya et al,**  **Clin Case Rep., 2021.** |
| ***NUP205*** |  |  |  |  |  |  |  |  |  |  |
|  | **c.3329T>C** | **p.Leu1110Pro** | **24 (hom)** | **1** | **SRNS (1 yrs)** |  |  |  |  | **Najafi M et al,**  **Front Pediatr., 2022.** |
|  | **c.5984T>C** | **p.Phe1995Ser** | **43 (hom)** | **2** | **SRNS (3 yrs),**  **FSGS on biopsy** |  | **ESKD (7 yrs)** | **kidney transplantation**  **(no disease recurrence)** | **CHD (bicuspid aortic valve, aortic insufficiency, aortic root enlargement)** | **Braun et al,  Nat. Genet., 2015** |
| ***NUP85*** |  |  |  |  |  |  |  |  |  |  |
|  | **c.405+1G>A**  **c.1741G>C** | **Donor splice site**  **p.Ala581Pro** | **5 (het)**  **17 (het)** | **2** | **SRNS (4, 7 yrs),**  **FSGS on biopsy** |  | **ESKD (7 yrs)** |  | **intellectual disability, short stature, partial GH deficiency** | **Braun et al,**  **J Clin Invest., 2018.** |
|  | **c.511 C > T**  **deletion of exon 2-5** | **p.Arg171Trp**  **p.Leu12_Gln135del** | **7 (het)**  **2-5 (het)** | **1** | **SRNS (3 yrs),**  **MCD on biopsy** |  |  |  |  | **Yang et al,**  **Pediatr Nephrol., 2025.** |
|  | **c.611T>A**  **c.1904T>G** | **p.Val204Glu**  **p.Leu635Arg** | **8 (het)**  **19 (het)** | **1** | **SRNS (3 yrs),**  **FSGS on biopsy** |  |  |  | **microcephaly, hypoplasia of the corpus callosum, simplified gyration, hypotonia, developmental delay, seizures** | **Gambadauro et al,**  **Genes (Basel), 2023.** |
|  | **c.1379G>A** | **p.Arg460Gln** | **14 (hom)** | **1** | **SRNS (10 yrs),**  **FSGS on biopsy** |  | **ESKD (15 yrs)** |  | **cleft lip-palate, mild intellectual disability** | **Kurt-Şükür et al,**  **Clin Genet., 2025** |
|  | **c.1430C>T** | **p.Ala477Val** | **15 (hom)** | **1** | **SRNS (8 yrs)** |  |  |  | **short stature** | **Braun et al,**  **J Clin Invest., 2018** |
|  | **c.1933C>T** | **p.Arg645Trp** | **19 (hom)** | **1** | **SRNS (11 yrs),**  **FSGS on biopsy** |  |  |  |  | **Braun et al,**  **J Clin Invest., 2018.** |
| ***NUP107*** |  |  |  |  |  |  |  |  |  |  |
|  | **c.303G>A** | **p.Met101Ile***  **(aberrant splicing)** | **4 (hom)** | **3** | **SRNS (6, 12, 14 yrs),**  **FSGS on biopsy** |  | **ESKD (17 yrs)** |  | **microcephaly, intellectual disability, short stature** | **Braun et al,**  **J Clin Invest., 2018.** |
|  | **c.303G>A** | **p.Met101Ile***  **(aberrant splicing)** | **4 (hom)** | **3** | **SRNS,**  **FSGS on biopsy** |  |  |  | **microcephaly, intellectual disability** | **Braun et al,**  **J Clin Invest., 2018.** |
|  | **c.303G>A** | **p.Met101Ile***  **(aberrant splicing)** | **4 (hom)** | **2** | **SRNS (10 yrs),**  **FSGS on biopsy** |  | **ESKD (14 yrs)** |  | **microcephaly, intellectual disability, arachnodactyly, high arched palate** | **Braun et al,**  **J Clin Invest., 2018.** |
|  | **c.303G>A** | **p.Met101Ile***  **(aberrant splicing)** | **4 (hom)** | **1** | **SRNS** |  | **ESKD (11 yrs)** |  | **microcephaly, intellectual disability, CHD (Ventricular septum defect), Nystagmus** | **Braun et al,**  **J Clin Invest., 2018.** |
|  | **c.303G>A** | **p.Met101Ile***  **(aberrant splicing)** | **4 (hom)** | **4** | **SRNS (4, 7 yrs),**  **FSGS on biopsy in 2 siblings,**  **no proteinuria in 2 siblings** |  | **ESKD (11 yrs)** | **kidney transplantation (16 yrs)** | **GAMOS (microcephaly, facial dysmorphism, intellectual disability)** | **Rosti et al, J Med Genet., 2017** |
|  | **c.303G>A** | **p.Met101Ile***  **(aberrant splicing)** | **4 (hom)** | **1** | **SRNS (12 yrs),**  **IgA nephropathy on renal biopsy** |  |  |  | **GAMOS (microcephaly, intellectual disability)** | **Rosti et al, J Med Genet., 2017** |
|  | **c.303G>A** | **p.Met101Ile***  **(aberrant splicing)** | **4 (hom)** | **3** | **SRNS (6, 12, 14 yrs),**  **FSGS on biopsy** |  | **ESKD (12 yrs)** |  | **microcephaly, intellectual disability** | **Bierzynska et al,**  **Kidney Int., 2017.** |
|  | **c.460A>G**  **c.1085C>T** | **p. Asp157Tyr**  **p. Ala362Val** | **6 (het)**  **13 (het)** | **1** | **SRNS (3 yrs)** | **resistant to CNI and MMF** | **ESKD (3 yrs)** |  | **microcephaly, seizures, neurodevelopmental delay, short stature** | **Pei et al,**  **Pediatr Res., 2025.** |
|  | **c.469G>T**  **c.2492A>C** | **p.Asp157Tyr**  **p.Asp831Ala** | **6 (het)**  **26 (het)** | **2** | **SRNS (10, 11 yrs),**  **FSGS on biopsy** |  | **ESKD (12 yrs)** |  |  | **Miyake,**  **Am J Hum Genet., 2015** |
|  | **c.580C>T**  **c.1199G>A** | **p.Arg194***  **p.Gly400Glu** | **7 (het)**  **14 (het)** | **1** | **SRNS (5 yrs),**  **FSGS on biopsy** |  |  |  |  | **Han et al,**  **Ital J Pediatr., 2024.** |
|  | **c.627_663dup37**  **c.2492A>C,** | **p.Leu225Phefs*15**  **p.Asp831Ala** | **7 (het)**  **26 (het)** | **1** | **SRNS (2 yrs),**  **FSGS on biopsy** |  | **ESKD (6 yrs)** |  |  | **Park et al,**  **Nephrol Dial Transplant., 2017.** |
|  | **c.627_663dup37**  **c.2492A>C,** | **p.Leu225Phefs*15**  **p.Asp831Ala** | **7 (het)**  **26 (het)** | **1** | **SRNS (3 yrs),**  **FSGS on biopsy** |  | **ESKD (7 yrs)** |  |  | **Park et al,**  **J Clin Med., 2020.** |
|  | **c.934delT**  **c.2492A>C** | **p.Tyr312Thrfs***  **p.Asp831Ala** | **11 (het)**  **26 (het)** | **1** | **SRNS (4 yrs),**  **FSGS on biopsy** |  | **ESKD (12 yrs)** |  |  | **Park et al,**  **J Clin Med., 2020.** |
|  | **c.969+1G>A**  **c.2492A>C** | **p.Asp831Ala** | **intron 11 (het)**  **26 (het)** | **1** | **SRNS (2 yrs),**  **FSGS on biopsy** | **resistant to CNI, CPA** | **ESKD (4 yrs)** |  |  | **Miyake,**  **Am J Hum Genet., 2015.** |
|  | **c.1079_1083del**  **c.2492A>C** | **p.Glu360Glyfs*6**  **p.Asp831Ala** | **12 (het)**  **26 (het)** | **2** | **SRNS (3 yrs),**  **Collapsing FSGS on biopsy** | **resistant to CNI, PLEX** | **ESKD (5 yrs)** |  |  | **Miyake,**  **Am J Hum Genet., 2015.** |
|  | **c.1079_1083del**  **c.2492A>C** | **p.Glu360Glyfs*6**  **p.Asp831Ala** | **12 (het)**  **26 (het)** | **2** | **SRNS (3 yrs),**  **FSGS on biopsy** | **resistant to CNI, CPA** | **ESKD (9 yrs)** |  |  | **Miyake,**  **Am J Hum Genet., 2015.** |
|  | **c.1079_1083del**  **c.2492A>C** | **p.Glu360Glyfs*6**  **p.Asp831Ala** | **12 (het)**  **26 (het)** | **2** | **SRNS (1, 4 yrs),**  **FSGS on biopsy** |  | **ESKD (2, 4 yrs)** |  |  | **Park et al,**  **Nephrol Dial Transplant., 2017.** |
|  | **c.1079_1083del**  **c.2492A>C** | **p.Glu360Glyfs*6**  **p.Asp831Ala** | **12 (het)**  **26 (het)** | **2** | **SRNS (2 yrs),**  **FSGS on biopsy** |  | **ESKD (2, 3 yrs)** |  |  | **Park et al,**  **Nephrol Dial Transplant., 2017.** |
|  | **c.1079_1083del**  **c.2492A>C** | **p.Glu360Glyfs*6**  **p.Asp831Ala** | **12 (het)**  **26 (het)** | **1** | **SRNS (2 yrs),**  **FSGS on biopsy** |  | **ESKD (5 yrs)** |  |  | **Park et al,**  **Nephrol Dial Transplant., 2017.** |
|  | **c.1079_1083del**  **c.2492A>C** | **p.Glu360Glyfs*6**  **p.Asp831Ala** | **12 (het)**  **26 (het)** | **1** | **SRNS (4 yrs),**  **FSGS on biopsy** |  | **ESKD (5 yrs)** |  |  | **Park et al,**  **Nephrol Dial Transplant., 2017.** |
|  | **c.1079_1083del**  **c.2492A>C** | **p.Glu360Glyfs*6**  **p.Asp831Ala** | **12 (het)**  **26 (het)** | **3** | **SRNS (2yrs),**  **MCD or FSGS on biopsy** | **resistant to CPA** | **ESKD**  **(7, 7, 10 yrs)** |  |  | **Miyake,**  **Am J Hum Genet., 2015.** |
|  | **c.1311 + 1G>A**  **c.1790C > T** | **Skipping of exon 15, in-frame deletion**  **p.Pro597Leu** | **intron 15 (het)**  **21 (het)** | **1** | **SRNS (8 yrs),**  **FSSG on biopsy** |  |  |  |  | **Yang et al,**  **Pediatr Nephrol., 2025.** |
|  | **c.1325G>A** | **p.Cys442Tyr** | **16 Hom** | **1** | **SRNS (2 yrs),**  **FSGS on biopsy** |  | **ESKD (2 yrs)** | **kidney transplantation**  **(no disease recurrence)** | **developmental delay** | **Bierzynska et al,**  **Kidney Int., 2017.** |
|  | **c.1360C>T**  **c.1695G>C** | **p.Arg454Trp**  **p.Lys565Trp**  **(skipping of exon 19, frame-shift)** | **16 (het)**  **19 (het)** | **1** |  |  | **ESKD (13 yrs)** |  |  | **Yang et al,**  **Pediatr Nephrol., 2025.** |
|  | **c.1735-3T>G**  **c.2492A>C** | **p.Asp831Ala** | **intron 20 (het)**  **26 (het)** | **2** | **SRNS (4, 5 yrs)** |  | **ESKD (5 yrs)** |  |  | **Park et al,**  **Nephrol Dial Transplant., 2017.** |
|  | **c.1735-3T>G**  **c.2492A>C** | **p.Asp831Ala** | **intron 20 (het)**  **26 (het)** | **1** | **SRNS (4 yrs),**  **FSGS on biopsy** |  | **ESKD (12 yrs)** |  |  | **Park et al,**  **J Clin Med., 2020.** |
|  | **c.2071C>T,**  **c.2492A>C** | **p.Gln691***  **p.Asp831Ala** | **23 (het)**  **26 (het)** | **2** | **SRNS (2, 4 yrs),**  **FSGS on biopsy** |  | **ESKD (6, 8 yrs)** |  |  | **Park et al,**  **Nephrol Dial Transplant., 2017.** |
|  | **c.2071C>T,**  **c.2492A>C** | **p.Gln691***  **p.Asp831Ala** | **23 (het)**  **26 (het)** | **1** | **SRNS (4 yrs)** |  | **ESKD (8 yrs)** |  |  | **Park et al,**  **J Clin Med., 2020.** |
|  | **c.2111delA**  **c.2350G>A** | **p.Lys704Serfs*10**  **p.Glu784Lys** | **24 (het)**  **25 (het)** | **1** |  |  | **ESKD (7 yrs)** | **kidney transplantation (8 yrs)** |  | **Pei et al,**  **Pediatr Res., 2025.** |
|  | **c.2564delC**  **c.2753C>T** | **p.Pro855fs*23**  **p.Pro918Leu** | **27 (het)**  **28 (het)** |  |  |  | **ESKD (10 yrs)** |  | **left ventricular hypertrophy** | **Han et al,**  **Ital J Pediatr., 2024.** |
|  | **c.2666A>G** | **p.Tyr889Cys** | **27 (hom)** | **1** | **SRNS (4 yrs),**  **DMS on biopsy** |  |  |  | **cleft lip, cleft palate** | **Braun et al,**  **J Clin Invest., 2018.** |
| ***NUP133*** |  |  |  |  |  |  |  |  |  |  |
|  | **c.182+387T>G**  **c.2898G>C** | **160 bp insertion in intron 1, p.Gly62Serfs*41**  **p.Lys966Asn** | **Intron 1**  **21 (het)** | **1** | **SRNS (1 yrs),**  **FSGS on biopsy** |  | **ESRD** |  |  | **Wang et al,**  **Clin Genet., 2023.** |
|  | **c.691C>G**  **c.3164T>C** | **p.Arg231Gly**  **p.Leu1055Ser** | **6 (het)**  **23 (het)** | **2** | **SRNS (9, 10 yrs),**  **FSGS on biopsy** |  | **ESKD  (13, 20 yrs)** |  |  | **Braun et al,**  **J Clin Invest., 2018.** |
|  |  |  |  |  |  |  |  |  |  |  |
|  | **c.2922T>G** | **p.Ser974Arg** | **21 (hom)** | **1** | **SRNS (3 yrs),**  **FSGS on biopsy** |  | **ESKD (6 yrs)** |  |  | **Braun et al,**  **J Clin Invest., 2018.** |
|  | **c.3335-11T>A** | **insertion of 9bp of intronic sequence between exon 25-26** | **intron 25** | **4** | **SRNS (1-2 years),** |  | **ESKD (1-3 yrs)** | **kidney transplantation (6-8 yrs) (no disease recurrence)** | **Galloway Mowat syndrome (microcephaly, intellectual disability, neurodevelopmental delay, convergent strabismus, muscle hypotonia)** | **Fujita et al,**  **Ann Neurol., 2018.** |
| ***NUP160*** |  |  |  |  |  |  |  |  |  |  |
|  | **c.1102-9T>G**  **c.4154C>T** | **abnormal splicing (partial deletion of  exon 8, insertion between exons 7 and 8)**  **p.Pro1385Leu** | **Intron 7 (het)**  **35 (het)** | **1** | **SRNS (22 yrs),**  **FSGS on biopsy** | **resistant to CNI** |  |  | **ovarian dysgenesis** | **Liu et al,**  **Clin Kidney J., 2024.** |
|  | **c.1179+5G>A** | **p.Phe368_Gln393del** | **Intron 8 (het)** | **2** | **SRNS**  **FSGS on biopsy** |  |  |  | **intellectual disability, seizures** | **Maddirevula et al,**  **Front Genet., 2020.** |
|  | **c.2241+1G>T**  **c.3656T> G** | **Skipping of exon 17, frame-shift**  **p.Leu1219Trp** | **17 (het)**  **31 (het)** | **1** | **SRNS (3 yrs),**  **FSGS on biopsy** | **resistant to CNI** | **ESKD (5 yrs)** | **kidney transplantation (7 yrs)** | **cord-like changes in the corpus uteri, mild intellectual disability, autism spectrum disorder** | **Yang et al,**  **Pediatr Nephrol., 2025.** |
|  | **c.2407G>A**  **c.2728C>T** | **p.Glu803Lys**  **p.Arg910*** | **19 (het)**  **22 (het)** | **2** | **SRNS (7, 16 yrs),**  **FSGS on biopsy** |  |  |  |  | **Braun et al,**  **J Clin Invest., 2018.** |
|  | **c.2407G > A**  **c.3330delA** | **p.Gln803Lys**  **p.Arg1110Argfs*128** | **19 (het)**  **28 (het)** | **1** | **SRNS (3 yrs),**  **MCD on biopsy** | **partial response to CNI** |  |  |  | **Yang et al,**  **Pediatr Nephrol., 2025.** |
|  | **c.2407G > A**  **c.3517C>T** | **p.Gln803Lys**  **p.Arg1173*** | **19 (het)**  **30 (het)** | **1** | **SRNS (7 yrs),**  **FSGS on biopsy** | **resistant to CNI** | **ESRD (15 yrs)** | **kidney transplantation (16 yrs)** |  | **Zhao et al,**  **J Am Soc Nephrol., 2019.** |

CHD, congenital heart disease; CNI, Calcineurin inhibitors; CPA; cyclophosphamide; DMS, diffuse mesangial sclerosis; ESKD, end stage kidney disease; FSGS, focal segmental glomerulosclerosis; GAMOS, Galloway-Mowat-syndrome; het, heterozygous; hom, homozygous; IgA, immunoglobulin A; MCD, minimal change disease; MMF, mycophenolate mofetil; PLEX, plasma exchange; PR, partial response; SRNS, steroid resistant nephrotic syndrome; yrs, years.

*the variant c.303G>A, p.Met101Ile* of *NUP107* leads to aberrant splicing with partial exon 4 skipping, resulting in a frame-shift mutation and reduction of the NUP107 transcript (Rosti et al, J Med Genet., 2017).

c. and p. changes refer to the following transcript variants:

*NUP93* (NM_014669.4); *NUP205* (NM_015135.2); *NUP85* (NM_024844.4); *NUP107* (NM_020401.3); *NUP133* (NM_018230.2), *NUP160* (NM_015231.1)
